# Design, Synthesis, and Anticancer Activity of a Selenium-Containing Galectin-3 and Galectin-9N Inhibitor

**DOI:** 10.3390/ijms23052581

**Published:** 2022-02-25

**Authors:** Sonia Di Gaetano, Luciano Pirone, Ioannis Galdadas, Serena Traboni, Alfonso Iadonisi, Emilia Pedone, Michele Saviano, Francesco Luigi Gervasio, Domenica Capasso

**Affiliations:** 1 Institute of Biostructures and Bioimaging, CNR, 80134 Naples, Italy; digaetan@unina.it (S.D.G.); luciano.pirone@cnr.it (L.P.); empedone@unina.it (E.P.); 2 Interuniversity Research Centre on Bioactive Peptides (CIRPEB), University of Naples “Federico II”, 80134 Naples, Italy; msaviano@unina.it; 3 Department of Chemistry, University College London, London WC1E 6BT, UK; ioannis.galdadas@unige.ch; 4 Institute of Pharmaceutical Sciences of Western Switzerland, University of Geneva, 1211 Geneva, Switzerland; 5 Department of Chemical Sciences, University of Naples Federico II, 80134 Naples, Italy; serena.traboni@unina.it (S.T.); iadonisi@unina.it (A.I.); 6 Institute of Crystallography, CNR, 70126 Bari, Italy; 7 Institute of Structural and Molecular Biology, University College London, London WC1E 6BT, UK; 8 School of Pharmaceutical Sciences, University of Geneva, 1211 Geneva, Switzerland; 9 Center for Life Sciences and Technologies (CESTEV), University of Naples “Federico II”, 80145 Naples, Italy

**Keywords:** galectin, digalactoside compounds, drug design, binding affinity melanoma cells

## Abstract

Galectins are soluble β-D-galactoside-binding proteins whose implication in cancer progression and disease outcome makes them prominent targets for therapeutic intervention. In this frame, the development of small inhibitors that block selectively the activity of galectins represents an important strategy for cancer therapy which is, however, still relatively underdeveloped. To this end, we designed here a rationally and efficiently novel diglycosylated compound, characterized by a selenoglycoside bond and the presence of a lipophilic benzyl group at both saccharide residues. The relatively high binding affinity of the new compound to the carbohydrate recognition domain of two galectins, galectin 3 and galectin 9, its good antiproliferative and anti-migration activity towards melanoma cells, as well as its anti-angiogenesis properties, pave the way for its further development as an anticancer agent.

## 1. Introduction

Galectins are a family of soluble β-D-galactoside-binding proteins characterized by a highly conserved carbohydrate recognition domain (CRD) [1] and are expressed in a broad range of animal species. These proteins are present in various cell types, including epithelial and endothelial cells from the skin, lung, and gut, as well as immune cells and fibroblasts [2,3,4]. Based on their molecular structure, galectins have been classified into three groups: (a) proto-galectins (galectin 1, -2, -5, -7, -10, -11, -13, -14 and -15) with a single CRD; (b) tandem repeat galectins (galectin 4, -6, -8, -9 and -12) with two distinct CRDs connected by a linker peptide; and (c) chimeric galectins represented by only one member, galectin 3, composed of a canonical domain connected to a long tail [5].

Galectins, synthesized on the cytoplasm, translocate rapidly to the extracellular space through non-classical exocytic pathways (non-ER/Golgi secretory route) [6], where they get exposed to a large variety of glycans. The binding of galectins to several carbohydrates attached to extracellular proteins and lipids and other membrane components leads to the formations of the so-called “galectin-glycoprotein lattice” [7], which controls the concentration, localization, and availability of glycosylated receptors on the cell surface. In that way, galectins modulate the transmembrane signaling events of diverse physiological and pathological processes, including among others cell–cell and cell–extracellular matrix interactions, cell adhesion, proliferation, differentiation, as well as angiogenesis [8,9], autoimmune processes [10], pulmonary fibrosis [11], cancer progression, and metastasis [12]. Moreover, galectins control infections by mediating the endocytosis of different pathogens through continuous surveying of the extracellular environment [13,14,15]. The role of galectins in all these cellular pathways would imply lack of specificity, but this is far from being the case, as the structural complexity and variability of the carbohydrates that galectins recognize result in specific interactions that allow galectins to selectively activate different pathways. 

Besides extracellular functions, intracellular galectins bind to several intracellular substrates to regulate different biological processes. As they do not interact with glycans, their activity is predominantly mediated by protein–protein interactions, although several carbohydrate molecules, such as lactose, are capable of inhibiting intracellular actions [16]. Galectins, in particular Gal-1 and Gal-3, have been shown to influence cell signaling through association with proteins such β-catenin and members of the RAS family [17]. Intracellular Gal-3 interacts with Bcl-2 and is able to block the intrinsic apoptotic pathway [18]. High intracellular Gal-3 levels are also known to affect the inflammatory response through various mechanisms [19]. 

As to cancer biology, galectins are highly involved in the regulation of apoptosis, tumor metastasis, tumor cell adhesion, migration, and angiogenesis [20]. Interestingly, the expression of galectins is altered in tumor cells compared to their normal counterparts. In many situations, this modified galectin expression correlates with the aggressiveness of tumors and the acquisition of the metastatic phenotype, indicating that galectins might modulate tumor progression and influence the disease outcome [21,22]. In particular, current literature data highlight the importance of the role of Gal-3 in melanoma progression [23,24]. Gordon-Alonso et al. [23] demonstrated that in melanoma with a dismal prognosis, Gal-3 binds the glycans of the extracellular matrix and of IFNγ, decreasing T-cell recruitment, which is the best predictor to date of patient survival [23]. A previous study reported that in melanoma cells, extracellular Gal-3 is able to induce the MMP-9 expression, activating the p38 MAPK pathway involved in cellular processes such as matrix degradation [24].

Therefore, the development of potent and selective small inhibitors that block the activity of galectins is a promising therapeutic approach for cancer. Accordingly, several natural or synthetic carbohydrates, able to bind galectins and interfere with their metabolism confirm the therapeutic potential of galectin inhibition. Simple lactose and N-acetyllactosamine (LacNAc) exhibit binding affinities in the high micromolar range, but their multivalent derivatives show greater affinity [25]. However, the use of these natural carbohydrates as drugs is impaired by their sensitivity to glucosidases, which reduce drastically their half-life in vivo. For this reason, specific derivatizations aimed at increasing their metabolic stability and specificity have been considered [26,27]. Among these, the presence of an alternative sulfur bridge in β-D-galactopyranosyl-(1-1)-thio-β-D-galactopyranoside (TDG) made the molecule stable to hydrolysis while retaining the same affinity to galectins as lactose and paved the way for the production of small symmetrical inhibitors with favorable properties [28]. TDG derivatives carrying different substitutions such as 4-aryl-1,2,3-triazolyl (TD139) [29], 3-benzamido [28], 4-amido-1,2,3-triazolyl [30], or 3-O-coumarylmethyl [31] moieties at the 3- and 3’-positions are able to bind Gal-1 and Gal-3 with nanomolar affinity. In particular, TD139 has recently been approved by the FDA for the treatment of idiopathic pulmonary fibrosis [31] and, 3,3′-O-dicoumarylmethyl substituted thiodigalactoside appears to be the first molecule capable of binding Gal-3 with selectivity over Gal-1 [31].

Recently, our research group has evaluated the antiproliferative effect of a series of sulfurated diglycosylated compounds against different human tumor cell lines [32]. In an attempt to establish a structure–activity relationship, we focused on the effect of both the saccharide residues and the number of bridging sulfur atoms (sulfide or disulfide structures) [32] on the antiproliferative properties and affinity of the different compounds against galectins. 

Here, we designed, synthesized, and characterized a new diglycosylated compound as a potential antitumor agent, characterized by a selenoglycoside bond and a suitably placed aromatic benzyl group at C-3 of both sugar residues. The choice of selenium as the bridging atom in place of sulfur was based on its chemical properties [33]. Selenium is an essential element for human metabolism and is a component of selenoamino acids that are necessary for numerous cellular functions. Organoselenides always sparked scientific interest for their anticancer and anti-inflammatory functions due to the duality of selenium which, unlike the lighter chalcogen, sulfur, has both antioxidant and peroxidant properties [33]. Ebselen, a glutathione peroxidase mimetic [34], was reported as anticancer [35,36], anti-inflammatory, antidepressant, and antibacterial [37], among others, and recently discovered as a potent inhibitor of the main protease from SARS-CoV-2 [38]. The poor solubility of the organoselenides that reduces their availability can be easily overcome by selenoglycosides [39,40], but the biological assessment of the latter has been limited to date by the need for expensive synthetic procedures. As a matter of fact, poor data are available on the bioactivity of selenoglycosides or 3,3′-substituted selenoglycosides derivatives covered by patent [41], as essentially are focused only on the assessment of their affinity towards lectin receptors [42] rather than their biological effect A recently introduced approach provided us with a very useful tool for quick production of digalactosyl selenides starting from a cheap selenenating agent [43], a method which is herein complemented by the availability of a simple synthetic strategy for the regioselective functionalization of carbohydrates with aromatic groups. 

The new compound herein described was evaluated for its ability to bind Gal-3^CRD^ and Gal-9N^CRD^ as well as for its antiproliferative, anti-migration, and anti-angiogenesis activity. Its relatively high affinity, anti-migration and anti-angiogenesis properties pave the way for further development of such compounds as antitumor agents.

## 2. Results and Discussion

### 2.1. Modelling and Molecular Docking

**Figure 1:**
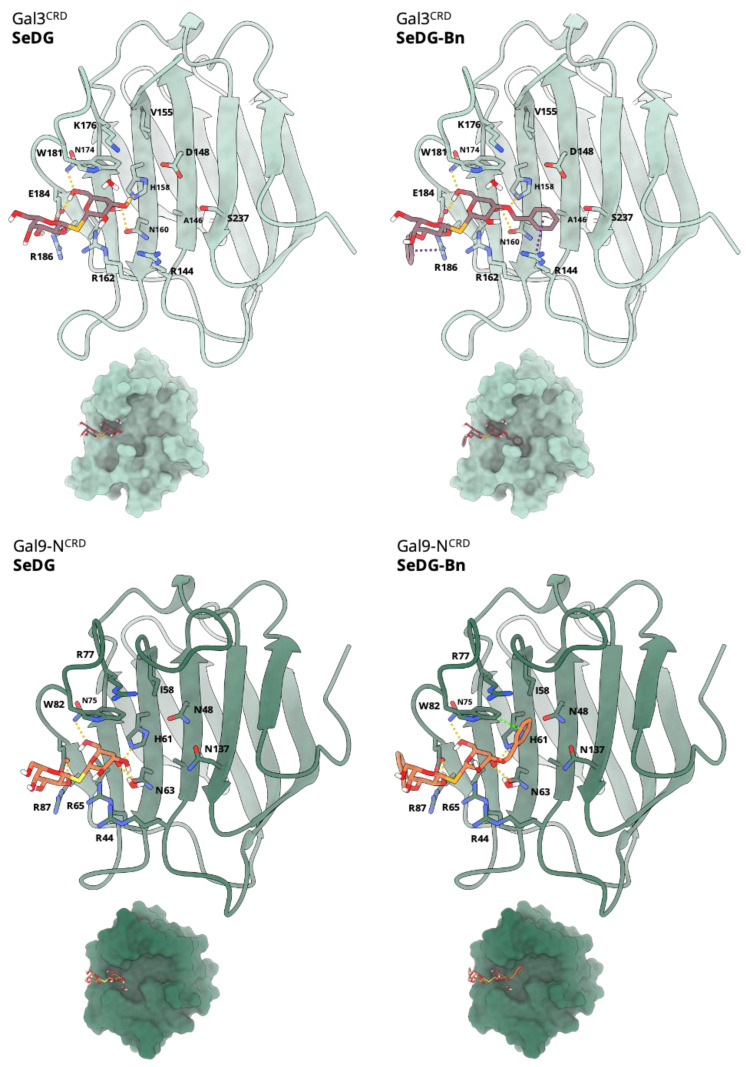
Docked poses of digalactosyl selenide (SeDG) and 3,3′-O-dibenzyl digalactosyl selenide (SeDG-Bn) to Gal-3^CRD^ and Gal-9N^CRD^. Hydrogen bonds between the side chains of the protein residues and the different ligands are depicted in dashed lines, π-cation interactions are depicted in purple dashed lines, and π-π interactions are depicted in green dashed lines.

Among the thiogalactosides described in the literature, we selected the simplest β-galactoside sugar, thiodigalactoside, and its selenated counterpart as lead compounds and we tried to further optimize their structures. Derivatization at position C3 has been proposed to improve the affinity for galectins through favorable interactions with charged residues [28]. Although derivatization at positions C4 and C6 is feasible, the hydrogen bond formed between the β-galactoside scaffold and galectins (Figure 1) indicate that these interactions not only stabilize the β-galactoside in the binding site but are expected to be necessary for substrate recognition. Consequently, we have focused on potential modifications only at position C3.

After comparison of several crystal structures of the CRD of Gal-3, and Gal-9N bound to different ligands, and given the presence of many arginines in the binding groove of the two galectins, we anticipated that arene substitutions in position C3 could enhance the affinity against specific galectins. Indeed, molecular docking of the digalactosyl selenide, SeDG, and its benzyl 3,3′-derivative, SeDG-Bn, to Gal-3, and Gal-9N indicates that the latter could bind more strongly to either galectin. In particular, in addition to the hydrogen bonds that the hydroxyl groups of the galactoside core form with the side chains of H158, N174, R162, E184, and W181, the presence of the phenyl rings in SeDG-Bn allows the formation of π-cation interactions with the side chains of R144 and R186 (Figure 1). Moreover, the relatively flexible benzyloxy group of SeDG-Bn is expected to be able to adopt an alternative conformation in both Gal-3^CRD^, and Gal-9N^CRD^, in which the phenyl ring forms a π-π interaction with W181. The enhanced binding of SeDG-Bn to Gal-3^CRD^, and Gal-9N^CRD^ due to the additional interactions is reflected to its better docking score (−7.92 and −7.72 kcal/mol, respectively) with respect to SeDG (−6.75 and −6.72 kcal/mol, respectively). Although it is known that the docking score of a compound alone does not necessarily correlate with its activity, the predicted binding pose of SeDG-Bn with respect to SeDG in combination with its better docking score prompted us to synthesize and evaluate the in vitro activity of SeDG-Bn.

### 2.2. Synthesis of Compounds

**Scheme 1:**
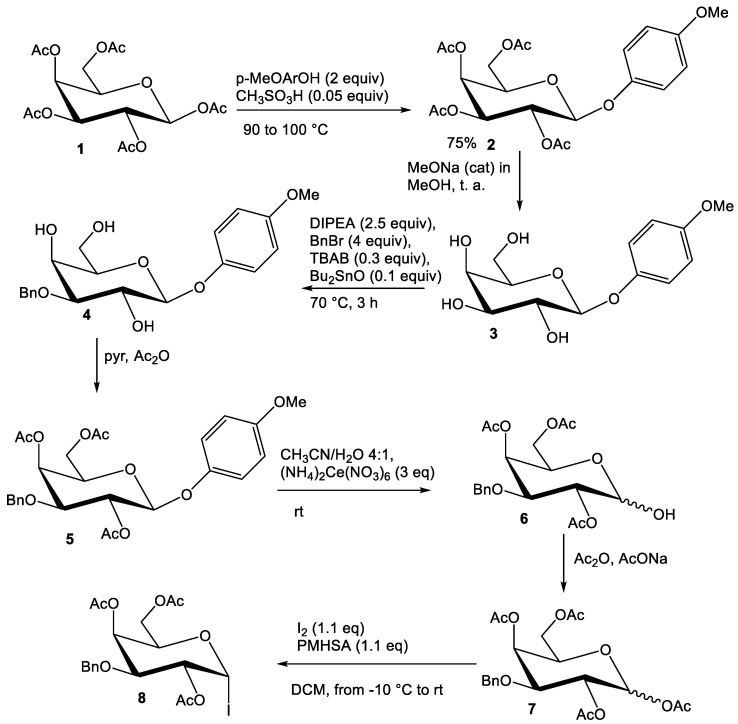
Synthesis of the key-intermediate **8**.

The synthesis of SeDG 10 started from the conversion of pentacetyl galactose **1** into the aryl glycoside **2**, through a recently described solvent-free glycosidation catalyzed by methansulfonic acid (Scheme 1) [44]. In this step, we introduced a para-methoxy-aryl as a suitable anomeric protecting group, which allowed the selective manipulation of the remaining reactive functionalities of the sugar. Compound **2** was smoothly deacetylated under standard Zemplén conditions (catalytic sodium methoxide in methanol) and the resulting tetrol 3 was submitted to a regioselective benzylation at O-3, following a recently established solvent-free methodology allowing the catalytic use of dibutyltin oxide [45]. The reaction provided 3-O-benzylated triol **4** in a good yield and excellent regioselectivity. After chromatographical purification, this compound was smoothly peracetylated under standard conditions, and the resulting derivative **5** was then deprotected at O-1 under oxidative conditions with cerium ammonium hexanitrate (CAN) in an acetonitrile/water mixture. The resulting anomeric hemiacetal **6** was acetylated with sodium acetate and acetic anhydride to yield compound **7** as an approximately equimolar anomeric mixture. The key step of the sequence was the synthesis of the diglycosyl selenide, which was performed by adapting a recently developed procedure, applied to date only to peracetylated precursors [43]. To this aim, compound **7** was first iodinated at the anomeric position with the iodine/silane combined reagent [45,46]. It is worth noting that such a reaction is usually performed at 40 °C (refluxing dichloromethane) with a peracetylated precursor, but the presence of an activating benzyl group made the substrate expectedly more reactive and, thus, a lower temperature could be used. The use of relatively milder conditions allowed us to minimize the undesired occurrence of a HI-mediated benzyl cleavage [47].

**Scheme 2:**
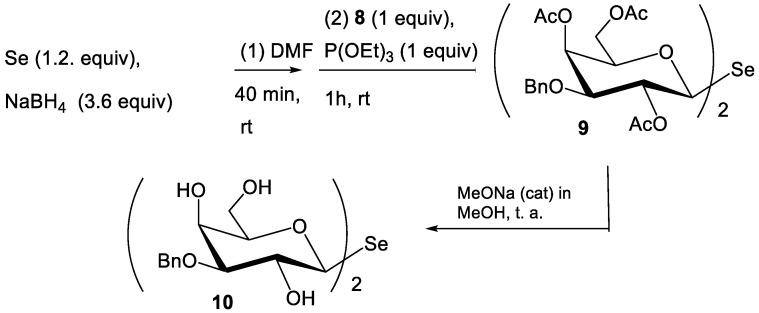
Final steps of the synthesis.

Glycosyl iodide **8** was isolated as a crude product after an extractive work-up and directly treated (Scheme 2) with a mixture of selenium and sodium borohydride, preliminarily kept under stirring for 40 min to favor the reduction of elementary selenium. In this step, triethyl phosphite was added to guarantee the chemoselectivity of the reaction, by minimizing the generation of the corresponding digalactosyl diselenide. The procedure provided selenide **9** in a satisfying overall yield (33% from **7**). After chromatographical purification, **9** was deacetylated under Zemplén conditions. Interestingly, application of the standard conditions (catalytic sodium methoxide at RT) led only to O-4 and O-6 deacetylation, and deprotection at the more congested O-2 site required more forced conditions in terms of both temperature and amount of NaOCH_3_. The deprotected selenide was eventually obtained in a satisfying yield (72%) after silica gel chromatography. All synthetic intermediates and the final product were characterized by NMR (Figure S1).

### 2.3. Binding Affinity Analysis of SeDG and SeDG-Bn to Gal-3^CRD^ and Gal-9N^CRD^

The genes encoding Gal-3^CRD^ (amino acid residues 112–250) and Gal-9N^CRD^ (amino acid residues 1–148) were cloned into the vector pETM11. *Escherichia coli* BL21(DE3)GOLD cells were transformed with recombinant constructs and the expression of the recombinant proteins were induced with 0.5 mM isopropyl β-D-1-thiogalactopyranoside (IPTG) for 16 h at 22 °C. Cells were harvested by centrifugation, suspended in 20 mM Tris⁄HCl, 500 mM NaCl, and 1 mM dithiothretiol pH 7.5. The proteins were purified by a two-step procedure consisting of a His-trap affinity chromatography and a gel filtration. The proteins were concentrated using a Centricon-10 ultrafiltration unit (Amicon, Merck, Darmstadt, Germany). The final yield was 20 mg/mL and 2 mg/mL for Gal-3^CRD^ and Gal-9N^CRD^, respectively. Gal-3^CRD^ and Gal-9N^CRD^, once expressed as recombinant proteins in a prokaryotic system and purified to homogeneity, were used to evaluate the binding of lactose, which is their natural substrate, to verify their functional integrity. Both proteins were fully active as they were able to bind lactose (Supplementary Figure S2).

**Figure 2:**
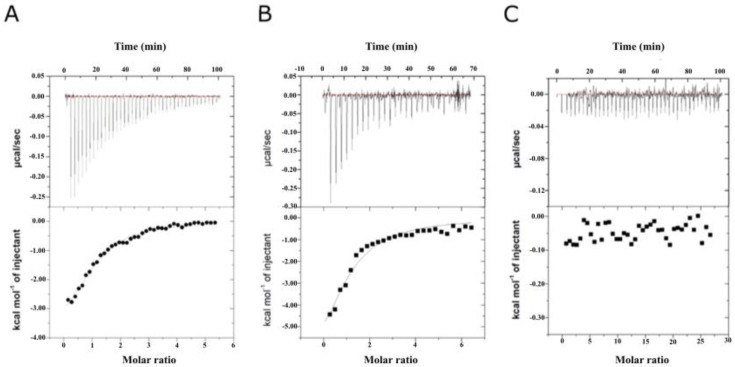
Interaction studies between SeDG and Gal-3^CRD^ or Gal-9N^CRD^ by ITC. Titration of (**A**) Gal-3^CRD^ and (**B**) Gal-9N^CRD^ with SeDG. Titration of (**C**) buffer with SeDG-Bn (negative control). The upper panels of the graphs depict the injections, while the lower panels depict the integrations of the peaks according to the molar ratio of the molecules tested.

**Figure 3:**
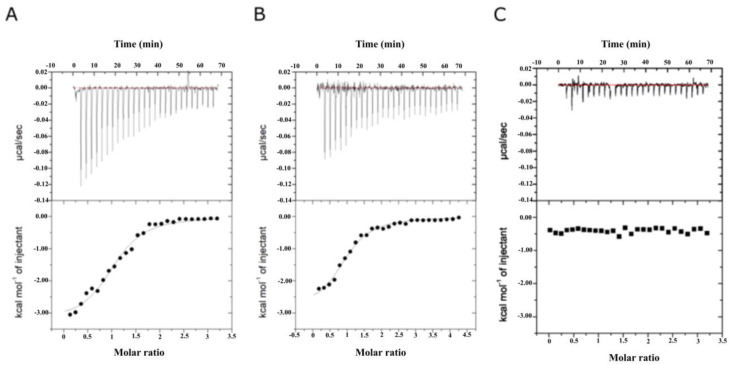
ITC binding studies of Gal-3^CRD^ and Gal-9N^CRD^ with SeDG-Bn. Titration of (**A**) Gal-3^CRD^ and (**B**) Gal-9N^CRD^ with SeDG-Bn. Titration of (**C**) buffer with SeDG-Bn (negative control). The top panels of the graphs correspond to the injections, while the bottom panels correspond to the integrations of the peaks according to the molar ratio of the tested molecules.

Both purified proteins were then probed for their binding affinity to SeDG and SeDG-Bn by ITC. The Kd values obtained for Gal-3^CRD^ and Gal-9N^CRD^ to SeDG resulted to be of 20.2 ± 2.2 µM and 22.4 ± 4.4 μM, respectively (Figure 2A–C and Table 1). On the contrary, the Kd values of SeDG-Bn were 2.2 ± 0.45 and 2.9 ± 0.3 µM for Gal-3^CRD^ and Gal-9N^CRD^, respectively, indicating the higher affinity of SeDG-Bn against both proteins (Figure 3 and Table 1).

The resulting values of Kd, ΔG, ΔH, −TΔS and *n* (stoichiometry) are summarized in Table 1. In all cases, the *n*-values indicate a 1:1 ratio of galectin/inhibitor. The negative ΔH suggests an exothermic reaction between the galectins and the ligands, while the positive ΔS suggests entropically driven reactions involving hydrophobic interactions, especially in the case of SeDG-Bn. The introduction of the benzyl group at position C3 of SeDG-Bn (Kd~2 μM) leads to a ~10-fold enhancement of the binding affinity for both proteins compared to the SeDG (Kd~20 μM), underlying the importance of the substitution. The enhanced binding affinity of SeDG-Bn to Gal-3^CRD^ and Gal-9N^CRD^ with respect to SeDG is reflected in a small change in the binding free energy (ΔΔGSeDG-Bn/SeDG of −1.38 kcal/mol and –0.4 kcal/mol, respectively).

### 2.4. Citotoxicity Assays

**Figure 4:**
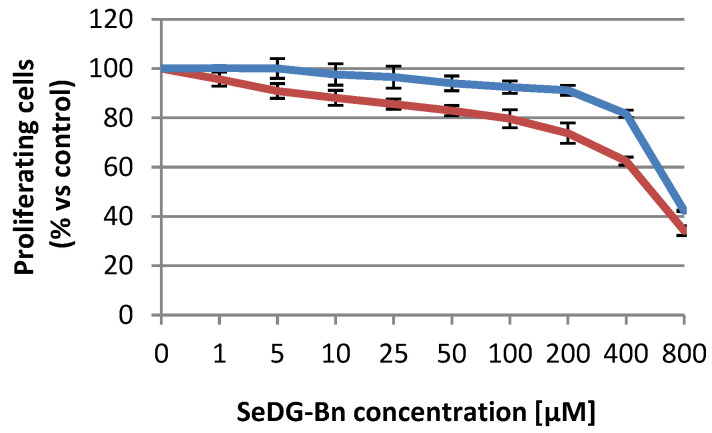
Dose–response curve on WM266 (red) and HDF (blue) cells. Cells were treated with SeDG-Bn at indicated concentrations and incubated for 48 h. The results are presented as the percentage of proliferating cells with respect to the control and are expressed as means ± SE of at least three independent experiments performed in triplicate. WM266: *p* < 0.05: at 6, 12, and 200 μM, *p* < 0.01 at other concentrations; HDF: *p* < 0.05 from 200 to 800 μM.

As previously reported, SeDG is unable to affect tumor cell proliferation even at 2 mM concentration [43]. In order to investigate the cellular effect of the aromatic derivatization at position C3 of both galactoside residues of SeDG-Bn, we performed a cytotoxicity assay on cell lines that differ in metastatic potential or histological origin. In particular, for this assay, we used a metastatic melanoma cell line (WM266), as well as low metastatic melanoma (A375), cervix carcinoma (HeLa), and human T lymphoblastoid cells (Jurkat). To verify the selectivity of action against cancer cells, we used the human dermal fibroblasts (HDF) as a healthy cellular model. First, we analyzed the cells to evaluate the presence of Gal-3 and Gal-9 and the results showed that all of them present significant amounts of one or both proteins (Supplementary Figure S3) [32]. We then incubated the cells in the presence of the SeDG and SeDG-Bn for 48 h with concentrations ranging from 1 to 800 μM. The most interesting result has been obtained with WM266 cells, in which the dose–effect curve (Figure 4) provides an IC50 of 430 μM for SeDG-Bn compared to 990 μM in healthy HDF cells demonstrating a selective activity towards cancer cells even though it ends up on the high micromolar range. Furthermore, very high values of IC50 (>900 µM) were obtained on other tested tumor cell lines treated with SeDG-Bn, demonstrating really scarce interference of SeDG-Bn with A375, HeLa, and Jurkat cell proliferation. The selective activity of SeDG-Bn towards WM266 cells suggests that SeDG-Bn is a good candidate for further optimization in melanoma therapy.

### 2.5. Cell Wound Healing and Invasion Assay

Several studies report that non-cytotoxic galectin inhibitors are able to interfere with galectin-mediated cancer development [48,49]. In in vivo experiments with nude mice, galectin inhibitors significantly reduced Gal-3 mediated lung metastasis [49]. Considering these previous data and encouraged by the bioactivity of SeDG-Bn on WM266 cells, we chose this tumor cell line to perform further biological assays. In particular, we evaluated the ability of SeDG and SeDG-Bn to inhibit WM266 cell migration and invasion, as well as angiogenesis progression carried out by means of endothelial cells [50,51].

Firstly, we performed an in vitro wound healing assay on WM266. To this aim, cell confluent monolayers were scratched and SeDG was added at 1 and 2.5 mM and SeDG-Bn at 100 and 200 µM concentrations. To avoid the cytotoxic effect of SeDG-Bn, in motility and invasion assays, we used concentrations lower than the IC50.

**Figure 5:**
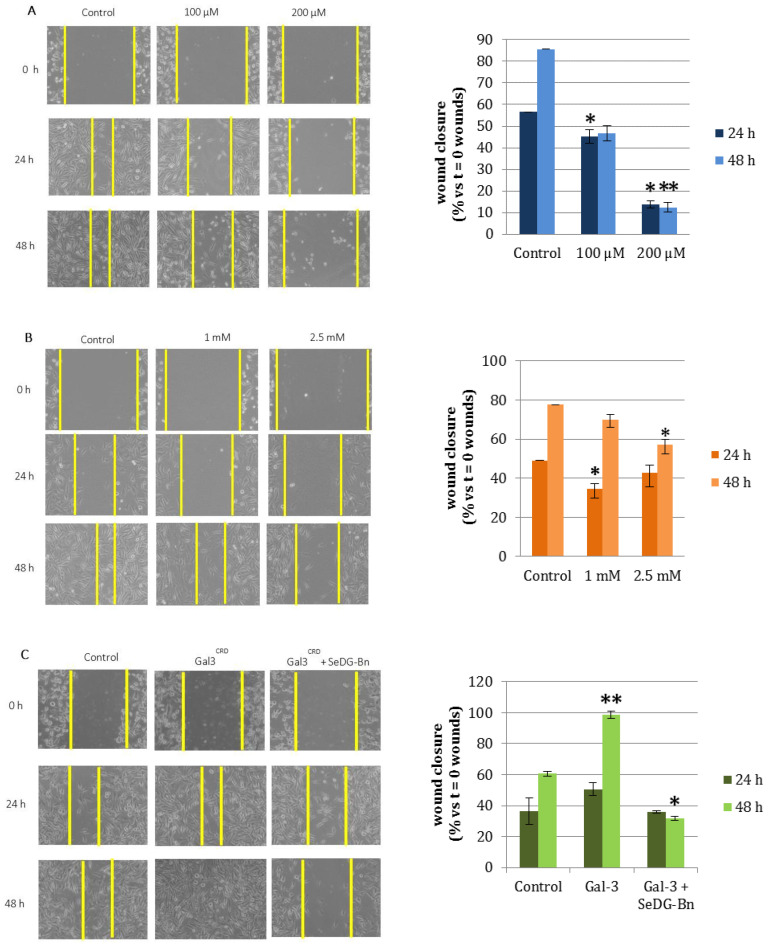
Cell wound healing assay. Effect of compounds on wound healing activity. WM266 cells were scratched and treated with (**A**) SeDG-Bn and (**B**) SeDG at the indicated concentrations. (**C**) WM266 cell after scratch in the presence of 50 µM Gal-3^CRD^ or 50 µM Gal-3^CRD^ pre-incubated with 200 µM SeDG-Bn. Each scratch area on the monolayer was photographed at 0, 24, and 48 h. The distance between the limits of the scratch was quantified and the size of wound closure was determined as follows: % wound closure = 1 − (wound width tx/wound width t0) × 100. Bars depict mean ± SE of three independent experiments, ** *p* < 0.01 * *p* < 0.05.

The results demonstrate that the wound healing of treated cells was significantly delayed compared to untreated cells. In particular, both compounds decreased the wound closure at all used concentrations and time intervals (Figure 5A,B). Notably, SeDG-Bn was able to inhibit cell migration at concentrations of an order of magnitude lower than SeDG did.

Under the same experimental conditions, the specificity of SeDG-Bn for Gal-3^CRD^ was validated using Gal-3^CRD^ as a cell motility stimulator in the absence or presence of SeDG-Bn (Figure 5C). Given the reported implication of Gal-3^CRD^ in cell migration [52], our results confirm that cells treated with 50 µM Gal-3^CRD^ migrate more than in the control. Noteworthy, Gal-3^CRD^-mediated wound closure was completely inhibited by SeDG-Bn treatment (Figure 5C). Such observation provides experimental evidence that SeDG-Bn can reduce cell migration by inhibiting the stimulating effect of Gal-3^CRD^.

**Figure 6:**
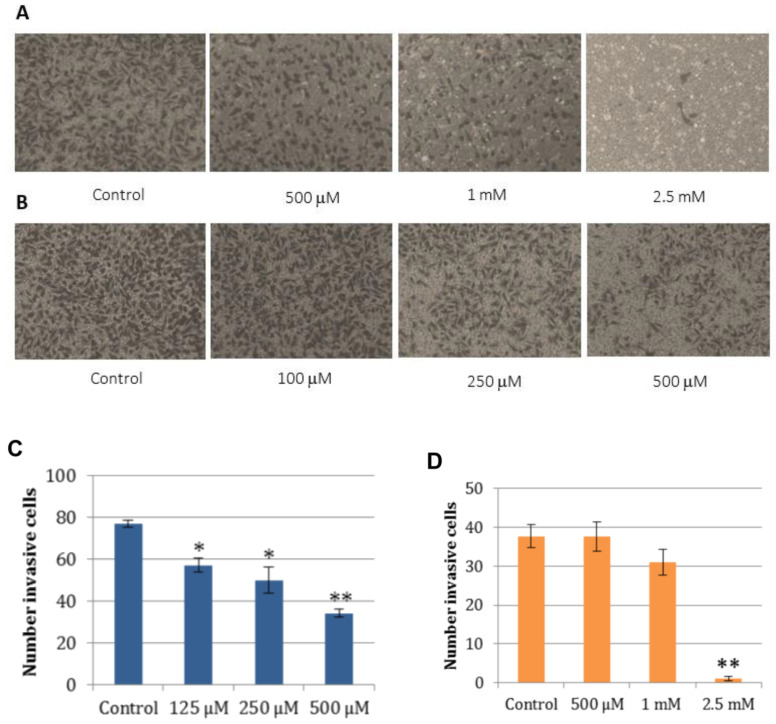
Inhibition of transwell invasion of WM266 cells by SeDG-Bn. WM266 cells were treated with (**A**) SeDG-Bn and (**B**) SeDG at the indicated concentrations and cells penetrating the matrix were fixed and stained after 24 h. Representative images of migrating cells stained with crystal violet are displayed at 10× magnification. Quantitative data of the invasion assay in the presence of (**C**) SeDG-Bn and (**D**) SeDG are reported in the graphs. Data represent the mean ± SE of three individual experiments. ** *p* < 0.01 * *p* < 0.05.

Then, we analyzed the ability of compounds to interfere with the invasion of WM266 cells throughout the extracellular matrix. The results obtained showed a marked inhibition of the WM266 invasion induced by the addition of SeDG-Bn acting in a dose-dependent mode (Figure 6). Moreover, SeDG was able to reduce the cells invasion but only at millimolar concentrations. 

Taken together, these results demonstrate that SeDG-Bn is able to interfere specifically with essential Gal-3^CRD^-driven cellular mechanisms involved in the metastatic cell diffusion, such as mobility and invasion of cancer cells.

### 2.6. Angiogenesis Assay

**Figure 7:**
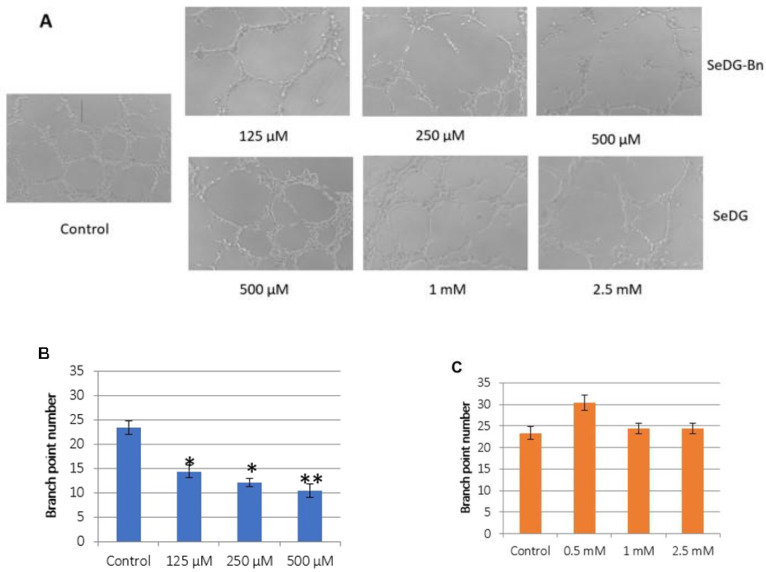
Capillary tube formation assay. (**A**) Representative images (10× magnification) of HuVec cells treated with SeDG-Bn and SeDG after 5 h of incubation at 37 °C. Quantitative analysis of branch point calculation for (**B**) SeDG-Bn and (**C**) SeDG, respectively. Data represent the mean ± SE of three individual experiments. ** *p* < 0.01 * *p* < 0.05.

Galectins have been shown to protect the cells from oxidative stress due to oxygen lack within the tumor microenvironment and act as pro-angiogenic receptors stimulating the growth of vascular endothelial cells [53,54,55]. Considering that melanoma requires high angiogenic activity [56], we evaluated the antiangiogenic activity of SeDG-Bn. To this aim, we used a common model of angiogenesis in which human umbilical vein cells (HuVeC) are layered on an appropriate matrix-forming cellular network (mesh-like structures). This model allowed us to verify the ability of digalactosyl selenides to inhibit cell vasculogenic capacity. Notably, SeDG-Bn was able to drastically prevent the formation of vessels at all of the evaluated concentrations in a dose-dependent manner, resulting in up to 60% reduction (Figure 7). In contrast, we found that SeDG at 2.5 mM results in a non-statistically significant change of capillary tube formation.

## 3. Materials and Methods

### 3.1. Molecular Modeling and Docking

Prior to docking calculations, the crystal structures of Gal-3^CRD^ (PDB ID 5H9P) and Gal-9N^CRD^ isoform (PDB ID 3WLU) were prepared using the Protein Preparation Wizard of the Schrödinger suite [57] in order to add bond orders and formal charges to the starting protein structures. The protonation state of each residue was determined using the PROPKA algorithm at pH 7.0 as implemented in Maestro.

Crystallographic water molecules that formed less than two hydrogen bonds with non-water atoms were removed, and the structure of each protein was relaxed using the OPLS3 force field [58]. Specifically, in the case of Gal-3CRD, the water molecule that was found to mediate an interaction between TD139 and W181 of Gal-3^CRD^ in the crystal structure (PDB ID 5H9P), and which was present also in the same position in other Gal-3^CRD^ structures (PDB IDs 3AYE, 4JC1, 4JCK), was considered to have a structural role and was maintained. Similarly, in the case of Gal-9NCRD, the water molecule that was found to mediate an interaction between R44, N63 and the co-crystallized ligand (PDB ID 3WLU) and that was found to mediate the same interaction in other Gal-9N^CRD^ structures (PDB IDs 2D6N, 2EAK) was maintained as well.

Se is not an element found frequently in drug-like molecules and, therefore, there are no standard parameters for it in many popular force fields, including OPLS3 which is the force filed used in the docking calculations of this study. Therefore, to get the necessary bonded and non-bonded parameters for the docking, we performed first principles calculations (with density functional) using the Force Field Builder tool of the Schrödinger suite, prior to the docking calculations. For instance, Force Field Builder ran density functional theory (DFT) calculations with the B3LYP/cc-pVTZ functional on SeDG and SeDG-Bn, which were then fitted to obtain the parameters for the torsions that include the Se atom, that are not part of the standard OPLS3 force field. The torsional energies were calculated by performing coordinate scans with Jaguar as implemented in the Schrödinger suite, followed by fitting the resulting energy curves to produce new force field parameters. 

To dock the SeDG and SeDG-Bn compounds to Gal-3CRD and Gal-9N^CRD^, we used the extra precision (XP) scoring function and docking protocol of Glide 7.6 (as implemented in the Schrödinger suite) [59]. Glide searches for favorable interactions between a ligand and a receptor. In our study, Glide was run with flexible ligand conformations, where multiple conformations of each ligand were generated before docking. Given the increased number of rotatable bonds in the proposed compounds and the conserved binding mode of the sugar rings that were found to be placed in the same position in all of the examined crystal structures for each system, we used positional constraints to enforce docked poses to place the sugar rings in a predefined volume of space relative to each receptor. Namely, we used the co-crystallized ligand of each receptor and defined a sphere of 1.8 Å into which the center of mass of each sugar ring would be forced to be placed. Moreover, although each receptor was treated as a rigid body during the docking process, the hydroxyl groups of S237 in Gal-3CRD, and S135 in Gal-9N^CRD^ were allowed to adopt different orientations with different ligands to produce the most favorable interactions. The introduction of these assumptions (i.e., positional constraints on the sugar rings and rotatable hydroxyl groups) was necessary for Glide to be able to reproduce the crystallographic poses of the co-crystallized ligand for each system. Figure 1 was generated using the ChimeraX visualization software [60].

### 3.2. Compound Synthesis

#### 3.2.1. Synthesis of **4**

Compound **3** (429 mg, 1.5 mmol), dibutiltin oxide (37 mg, 0.15 mmol), and tetrabutylammonium bromide (TBAB) (145 mg, 0.45 mmol) were weighted in a round-bottomed vessel. Diisopropyl amine (DIPEA) (0.65 mL, 3.7 mmol) and benzyl bromide di (0.71 mL, 6.0 mmol) were sequentially added to the mixture of solids, and the vessel was placed in an oil bath at 70 °C. After three hours, the mixture was concentrated in vacuo and the residue loaded on the top of a silica-gel column. Elution with ethyl acetate yielded compound **4** as an oil (355 mg, 63% yield). 

^1^H NMR (400 MHz, DMSO-d) δ 7.45–7.10 (benzyl aromatic H), 6.96 (2H, d, J = 9.2 Hz, p-methoxyaryl aromatic H), 6.83 (2H, d, J = 9.2 Hz, p-methoxyaryl aromatic H), 5.30 (1H, d, J = 5.2 Hz, –OH), 4.71 and 4.55 (2H, AB, J = 12.4 Hz, –CH_2_Ph), 4.77 (1H, d, J = 7.6 Hz, H-1), 4.66 (1H, t, J = 4.8 Hz, 6-OH), 4.61 (1H, d, J = 5.6 Hz, –OH), 3.97 (1H, m, H-4), 3.72 (1H, m, H-2), 3.70 (3H, s, –OCH_3_), 3.55 (1H, m, H-5), 3.48 (2H, m, H_2_-6), 3.33 (1H, dd, J = 3.2 and 10.0, H-3). ^13^C NMR (400 MHz, CDCl_3_) δ 154.6, 151.8, 139.4, 128.4, 127.8, 127.5, 118.0, 114.7, 102.4, 101.5, 75.6, 70.5, 69.7, 64.8, 60.6, 55.7.

#### 3.2.2. Synthesis of **5**

Acetic anhydride (1.5 mL) was added to a solution of compound **2** in (350 mg, 0.93 mmol) in pyridine (3 mL). The mixture was left overnight at RT and then methanol (ca 1 mL) was added to the mixture kept in a cool bath. The mixture was diluted with dichloromethane and transferred into a separatory funnel. The organic phase was washed with sat sodium carbonate and the aqueous phase re-extracted with dichloromethane. Combined organic phases were dried with anhydrous sodium sulfate, filtered, and concentrated in vacuo to yield pure compound 5 as an oil (600 mg, quantitative yield).

^1^H NMR (400 MHz, CDCl_3_) δ 7.40–7.15 (benzyl aromatic H), 6.92 (2H, d, J = 9.2 Hz, p-methoxyaryl aromatic H), 6.79 (2H, d, J = 9.2 Hz, p-methoxyaryl aromatic H), 5.55 (1H, bd, J = 3.6 Hz, H-4), 5.37 (1H, dd, J = 8.0 and 10.0 Hz, H-2), 4.80 (1H, d, J = 8.0 Hz, H-1), 4.71 and 4.43 (2H, AB, J = 12.4 Hz, –CH_2_Ph), 4.20 (2H, bd, J = 6.4 Hz, H2-6), 3.90 (1H, bt, J = 6.4 Hz, H-5), 3.75 (3H, s, –OCH_3_), 3.60 (1H, dd, J = 3.6 and 10.0 Hz, H-3), 2.18, 2.08, 2.05 (9H, 3 × s, 3 × –COCH_3_). ^13^C NMR (400 MHz, CDCl_3_) δ 170.3 (×2), 169.2, 155.4, 151.1, 137.3, 128.2, 127.7, 125.2, 118.4, 114.3, 100.7, 76.4, 71.2, 71.0, 70.2, 65.6, 61.8, 55.5, 20.7.

#### 3.2.3. Synthesis of **6**

Ceric ammonium hexanitrate (CAN) (340 mg, 1.44 mmol) was added to a solution of compound **5** (312 mg, 0.48 mmol) in 4:1 acetonitrile/water (5 mL). The mixture was kept under stirring at RT for 30 min, then diluted with ethyl acetate and transferred into a separatory funnel, washing the vessel with additional amounts of water and ethyl acetate. The organic phase was separated and the aqueous phase re-extracted with further ethyl acetate. The combined organic phases were dried with anhydrous sodium sulfate, filtered, and concentrated in vacuo. The residue was purified by silica-gel flash chromatography (eluent: hexane/ethyl acetate from 1:1 to 0:1) to yield hemiacetal compound **6** as an oil (150 mg, yield 78%).

Anomeric mixture α/β ca 5:1. ^1^H NMR (400 MHz, CDCl_3_) signals of prevalent α-anomer at δ 7.40–7.15 (benzyl aromatic H), 5.57 (1H, bd, J = 3.2 Hz, H-1), 5.44 (1H, bd, J = 2.8 Hz, H-4), 5.05 (1H, dd, J = 3.2 and 10.4 Hz, H-2), 4.70 and 4.45 (2H, AB, J = 11.6 Hz, –CH_2_Ph), 4.20–4.00 (2H, m, H_2_-6), 3.98 (1H, dd, J = 3.2 and 10.4 Hz, H-3), 3.82 (1H, bt, J = 6.8 Hz, H-5), 2.12, 2.06, 2.05 (9H, 3 × s, 3 × –COCH_3_). ^13^C NMR (400 MHz, CDCl_3_) signals of prevalent α-anomer at δ 170.6, 170.4 (×2), 137.5, 128.2, 127.7, 90.5, 72.6, 71.6, 70.1, 67.3, 66.4, 62.3, 20.7, 20.6 (×2).

#### 3.2.4. Synthesis of **7**

Acetic anhydride (0.7 mL) was added to a vessel containing hemiacetal **6** (140 mg, 0.36 mmol) and sodium acetate (10 mg, 0.060 mmol).The mixture was kept under stirring at 120 °C (oil bath) for 30 min, then diluted with dichloromethane and transferred into a separatory funnel. The organic phase was washed with sat aq carbonate, and the aqueous phase re-extracted with further dichloromethane. The combined organic phases were dried with anhydrous sodium sulfate, filtered, and concentrated in vacuo to yield compound **7** (152 mg, 97%) directly used in the following step.

^1^H NMR (400 MHz, CDCl_3_) δ 7.40–7.15 (benzyl aromatic H), 6.33 (1H, d, J = 3.6 Hz, H-1 α), 5.60 (1H, bd, J = 2.0 Hz, H-4 α), 5.58 (1H, d, J = 8.4 Hz, H-1 β), 5.52 (1H, bd, J = 2.0 Hz, H-4 β), 5.25–5.15 (2H, overlapped signals, H-2 α and β), 4.72-4.30 (4H, 2 × AB, J = 11.6 Hz, –CH_2_Ph α and β), 4.24 (1H, bt, J = 7.2 Hz, H-5 α), 4.20-4.00 (4H, overlapped signals, H_2_-6 α andβ), 3.93 (1H, bt, J = 7.2 Hz, H-5 β), 3.90 (1H, dd, J = 3.6 and 10.4 Hz, H-3 α), 3.60 (1H, dd, J = 3.6 and 10.4 Hz, H-3 β), 2.13, 2.12, 2.09, 2.07, 2.04, 2.01, 1.99, 1.98 (8 × –COCH_3_ α and β). ^13^C NMR (400 MHz, CDCl_3_) δ 170.3–168.8, 138.3, 137.3, 128.4-127.2, 92.0, 89.8, 76.4, 72.8, 71.8, 71.7, 71.6, 69.3, 69.2, 68.9, 68.2, 66.5, 65.6, 61.7, 20.6.

#### 3.2.5. Synthesis of **8**

To a solution of **7** (145 mg, 0.33 mmol) and I_2_ (67 mg, 0.26 mmol) in anhydrous DCM (4 mL), triethylsilane (42 µL, 0.26 mmol) was added at 0 °C. After 5 min, TLC analysis of the mixture (eluent: hexane/ethyl acetate 3:2) proves the nearly complete conversion of 7 into a less polar and very UV-visible product. After adding solid NaHCO_3_ (70 mg), the cool mixture was diluted with dichloromethane and the organic phase was washed with aq. sodium carbonate containing sodium thiosulfate (the latter is added portionwise as a solid until consumption of residual iodine indicated by discoloration of the organic phase upon shaking). The organic phase was then washed with water, dried, and concentrated. The crude residue was directly adopted for the subsequent selenoglycosidation steps.

#### 3.2.6. Synthesis of **9**

A mixture of elementary selenium (32 mg, 0.40 mmol) and sodium borohydride (45 mg, 1.2 mmol) was suspended in DMF (1 mL) and the suspension was kept under stirring at RT. After 40 min, triethyl phosphite (58 µL, 0.40 mmol) was added (with an instantaneous discoloration) and the mixture was poured into a vessel containing crude glycosyl iodide **8** (0.33 mmol). The vessel adopted for the first step was washed with portionwise DMF (1 mL overall). The mixture was kept under stirring at room temperature for one hour. The reaction was quenched with acetic acid (0.3 mL), and the mixture was transferred into a separatory funnel and diluted with dichloromethane. The organic phase was washed with aq sodium carbonate and the aqueous phase re-extracted with dichloromethane. Combined organic phases were dried with sodium sulfate, filtrated and concentrated. The residue was purified by silica-gel flash chromatography (eluent: hexane/ethyl acetate from 3:2 to 0:1) to yield compound **9** (46 mg, 33% yield).

^1^H NMR (400 MHz, CDCl_3_) δ 7.40–7.15 (benzyl aromatic H), 5.55 (1H, bd, J = 3.2 Hz, H-4), 5.19 (1H, t, J = 10.0 Hz, H-2), 4.94 (1H, d, J = 10.0 Hz, H-1), 4.69–4.39 (2H, AB, J = 12.4 Hz, –OCH_2_Ph), 4.20–4.05 (2H, m, H_2_-6), 3.74 (1H, bt, J = 6.8 Hz, H-5), 3.54 (1H, dd, J = 3.6 and 9.6 Hz, H-3), 2.14, 2.04, 2.00 (9H, 3 × s, 3 × –COCH_3_). ^13^C NMR (100 MHz, CDCl_3_) δ 170.2 (×2), 169.6, 137.2, 128.3, 127.7, 76.0, 75.8, 71.3, 69.5, 66.0, 61.9, 20.7. Elementary analysis calcd for C_38_H_46_O_16_Se: C 57.48, H 5.53; found C 57.40, H 5.55. HRMS *m/z* calcd for C_38_H_46_O_16_Se [M + Na]+ = 861.1849; found: 861.1840.

#### 3.2.7. Synthesis of **10** (SeDG-Bn)

To a solution of compound **9** (24 mg, 0.028 mmol) in MeOH (1 mL), a solution of sodium methoxide in methanol (5.6 µmol) was added, preliminarily prepared by adding a weighted amount of NaH (60% suspension) in methanol. The mixture was left under stirring at RT until the detection via TLC (eluent: chloroform/methanol 9:1) of a single product. The mixture was treated with Amberlyst H+ resin (preliminarily washed with methanol) until neutrality. The supernatant was transferred to another vessel, where it was concentrated in vacuo. NMR of the residue evidenced a large prevalence of a partially deprotected product still acetylated at O-2. The residue was then dissolved again with methanol and treated with sodium methoxide in methanol (0.028 mmol) 75 °C for 30 min. The mixture was treated with Amberlyst H+ resin (preliminarily washed with methanol) until neutrality. The supernatant was transferred to another vessel, where it was concentrated in vacuo. The residue was purified by silica-gel flash chromatography (eluent: chloroform/methanol from 10:1 to 8:1) to yield pure SeDG-Bn (12 mg, 72% yield). ^1^H NMR (400 MHz, D_2_O) δ 7.50–7.20 (benzyl aromatic H), 4.90 (1H, d, J = 10.0 Hz, H-1), 4.67–4.54 (2H, AB, J = 12.4 Hz, –OCH_2_Ph), 4.04 (1H, bs, H-4), 3.68 (1H, t, J = 10.0 Hz, H-2), 3.65–3.42 (4H, overlapped signals, H-3, H-5 and H_2_-6). ^13^C NMR (100 MHz, D_2_O) δ 137.1 (aromatic ipso C), 128.4–128.2 (aromatic CH), 80.8 (C-1), 80.1 (×2) (C-3 and C-5), 71.0, 69.3 (benzyl CH_2_ and C-2), 65.5 (C-4), 61.1 (C-6). Elementary analysis calcd for C_26_H_34_O_10_Se: C 53.34, H 5.85; found C 53.45, H 5.80. HRMS *m/z* calcd for C_26_H_34_O_10_Se [M + Na]+ = 609.1215; found: 609.1207.

### 3.3. Gal-3^CRD^ and Gal-9N^CRD^ Expression and Purification

Genes encoding the CRD of Gal-3 and Gal-9N, corresponding to residues 112–250 and 1–148, respectively, were purchased from Genewiz. The two proteins were cloned into pETM11 expression vector with a N-terminal polyHis-tag that is removable by TEV protease. The recombinant vectors pETM11-gal3^CRD^ and pETM11-gal9^CRD^ were used to transform *E. coli* BL21(DE3)GOLD cells and a single colony was grown overnight at 37 °C in 20 mL of LB medium. In total, 10 mL of the saturated cultures were transferred in 1 L of LB medium and grown at 37 °C to 0.8 OD 600 nm before induction with IPTG (0.5 mM). The inductions were kept for 16 and for 3 h at 22 °C in the expression of Gal-3^CRD^ and Gal-9N^CRD^, respectively. The cells were harvested by centrifugation, re-suspended in 30 mL of lysis buffer (20 mM NaP, 500 mM NaCl, 10 mM imidazole pH 7.0 for Gal-3^CRD^ and pH 7.5 Gal-9N^CRD^), and sonicated. After centrifugation (30,000× *g*, 30 min, 4 °C) the soluble fraction was subjected to affinity chromatography onto an HisTrapTM column (5 mL, GE Healthcare) previously equilibrated in lysis buffer. Elution was carried out with 300 mM imidazole. Fractions containing the recombinant protein were, finally, applied onto a Superdex 75 10/300 column (GE Healthcare), connected to an AKTA system (GE Healthcare) pre-equilibrated in 20 mM NaP, 150 mM NaCl pH 7.0 for the purification of Gal-3^CRD^ and pH 7.5 for Gal-9N^CRD^.

### 3.4. Isothermal Titration Calorimetry

The purified proteins were used to perform interaction studies. The interaction between the molecule SeDG-Bn with Gal-3^CRD^ or Gal-9N^CRD^ was tested by isothermal titration calorimetry (ITC) experiments, which were carried out on an ITC200 calorimeter (MicroCal/GE Healthcare) at 25 °C. SeDG or SeDG-Bn (500 µM) were titrated into a solution of Gal-3^CRD^ or Gal-9N^CRD^ (20 µM). Before all titrations, molecules were dissolved in 20 mM NaP, 150 mM NaCl pH 7.0 (when the molecules were assayed against Gal-3^CRD^) or pH 7.5 (for Gal-9N^CRD^). The sample cell was filled with 280 μL of Gal-3^CRD^ or Gal-9N^CRD^. Molecules were injected in a volume of 0.2 μL for the first injection and 1 μL for the next 39 injections using an injection interval of 150 s. The control experiment, obtained by injection of the same amount of each molecule in buffer solution, was subtracted to correct for heat production upon molecules dilution. All data were analyzed and fitted using the Microcal Origin version 7.0 software package. Binding enthalpy, dissociation constants, and stoichiometry were determined by fitting the data using a one-set-of-site-binding model [61].

### 3.5. Cell Lines and Culture Conditions

Human adenocarcinoma cell line (HeLa), human low metastatic melanoma (A375), human metastatic melanoma (WM266), and human T lymphoblastoid cell line (Jurkat) were from ATCC (Manassas, VA, USA); normal human dermal fibroblasts (HDF) were kindly provided by Dr Annalisa Tito (Arterra, Biosciences, Naples, Italy). HeLa, A375, and HDF were grown in DMEM supplemented with 10% fetal bovine serum (FBS), 1% glutamine, 100 U/mL penicillin, and 100 µg/mL streptomycin (Euroclone, Milano, Italy). Under the same experimental conditions, WM266 and Jurkat were grown in RPMI supplemented with heat-inactivated 10% fetal bovine serum (FBS), 2.5 mM glutamine, 100 U/mL penicillin, and 100 µg/mL streptomycin (Euroclone). Human umbilical vein endothelial cells (HuVec) were purchased from Lonza. They were grown in EBM2 basal medium enriched with Single Quot Supplements. All the cells were maintained in humidified air containing 5% CO_2_, at 37 °C.

### 3.6. Western Blotting

Cell lysates were obtained incubating human tumor cells in a lysis buffer (50 mM Hepes pH 7.4, 50 mM NaCl, 1% Triton) supplemented with protease inhibitor cocktail (Roche, Basilea Switzerland) for 30 min on ice and centrifuged at 13,000 rpm for 30 min. The protein concentrations were determined by Bradford method using Bio-Rad reagent (Bio-Rad Laboratories, Hercules, CA, USA). The obtained lysates were loaded on a SDS-polyacrylamide gel electrophoresis and transferred to a PVDF membrane (Bio-Rad). Successively, the membrane was incubated with primary antibodies (anti-Gal-3 and anti-Gal-9 from R&D, anti-actin from Sigma Aldrich, St. Louis, MO, USA) 1 h at RT. Actin was used as an internal standard. Bands were visualized with LiteAblot (Euroclone, Pero, Italy) chemiluminescence as substrate and the signal was acquired with ChemiDoc XRS System (Bio-Rad) and analyzed with the QuantityONE software. 

### 3.7. Cell Proliferation Assay

Cells were plated at a density of 1200 cells/well for HeLa, 1000 for A375, 10,000 for Jurkat and 2000 for WM266 and HDF in 96-well microplates (Thermofisher, Waltham, MA, USA). After 24 h incubation, cells were treated with increasing concentrations of synthetized compounds for 48 h. Cell proliferation was determined by using MTT (Sigma Aldrich, St. Louis, MO, USA) for adherent cells [62]. In the case of Jurkat that are suspension lymphoblasts, the proliferation was investigated by CCK-8 assay [63]. Plates were then analyzed by using a microplate reader (Enspire, Perkin Elmer, Waltham, MA, USA) at 570 nm for MTT and at 450 for CCK-8. The mean value of proliferating cells for each treatment was compared to untreated cells (control). All experiments were performed at least in triplicate and repeated at least 3 times. The IC50 values were calculated using Graph-Pad Prism (version 6.01, Graph-Pad software Inc., San Diego, CA, USA).

### 3.8. The Wound Healing Assay

Analysis of cell migration in vitro has been carried out by using the wound healing scratch assay. WM266 cells were grown until they reached a confluent monolayer. After, they were linearly scratched with a pipette tip to generate a cell-free space. The detached cells were removed with PBS and digalactosyl selenides at indicated concentrations or 50 µM Gal-3^CRD^ or 50 µM Gal-3^CRD^ pre-incubated with 200 µM SeDG-Bn for 5 min, were added to the adherent cells at different concentrations in RPMI, then cells were incubated at 37 °C and successively the scratch area was photographed at 0, 24, and 48 h [64]. The area related to each incision was photographed at the time 0, 24, and 48 h using a phase contrast optical microscope with a 10× magnification (Zeiss). The distance between the edges of the incisions was measured by AxioVision Rel. 4.8 software and its mean value was determined as follows: wound closure (%) = 1 − (wound width tx/wound width t0) × 100. Bars depict mean ± SE of three independent experiments. 

### 3.9. Invasion Assay

To analyze cell invasion, the surface of a filter membrane of a transwell insert was coated with extracellular matrix (ECL) (Millipore). Cells (5 × 10^4^) suspended in 100 μL of serum-free medium were incubated with various concentrations of molecules and successively they were seeded onto the upper compartment of the transwell, whereas the lower chambers were filled with medium containing 10% FCS [65]. After 24 h, the cells were fixed with 10% formalin and stained with crystal violet. The remaining cells in the upper side of the transwell were removed and cells on the undersurface of the filters, the invaded cells, were visualized and counted from six randomly selected fields (10× magnification) under a phase-contrast microscope with AxioVision Rel. 4.8 software.

### 3.10. Angiogenesis Assay

Angiogenesis assay was performed by using “In Vitro Angiogenesis Assay Kit” (Merck, Burlington, MA, USA). Briefly, 100 μL of ECL were plated in a pre-cooled 96-well plate and incubated for 1 h at 37 °C. 10,000 cells (HuVec), suspended in EGM-2 [66], incubated with digalactosyl selenides, and seeded onto solidified matrix, according to manufacturer’s instructions. After 5–6 h of incubation at 37 °C, the tube formation was examined by means of a phase contrast optical microscope (Axiovert 200 Zeiss) at 10× magnification and the branch point number was calculated from 3 random fields per each treatment. Imagines were acquired by AxioVision Rel. 4.8 software.

### 3.11. Statistical Data Analysis

All data are presented as mean values ± SE. The statistical analysis was performed using Student’s t-test, unpaired, two-sided, *p* < 0.05 was considered significant.

## 4. Conclusions

Although the involvement of galectins in many diseases such as diabetes, obesity, atherosclerosis, autoimmune diseases, and cancer has been ascertained, many aspects of the complex metabolic networks in which they are involved are still unknown and their therapeutic potential has not been exploited.

Recent studies have reported high expression of Gal-3 and Gal-9 in different tumors such as glioma [67,68], ovariancarcinoma [69], pancreatic ductal adenocarcinoma [70], chronic lymphocytic leukemia [71], and melanoma [23]. Given the implication of Gal-3 and Gal-9 in metabolic pathways that regulate tumor progression, we designed and synthesized a new molecule, SeDG-Bn, able to bind Gal-3^CRD^ and Gal-9N^CRD^ and affect tumor cell mobility, invasion, and angiogenesis. We started from a selectively functionalized digalactosyl selenide; thus, selecting a bridging selenium atom between the sugars in order to exploit the interesting in vivo properties of selenium. For this purpose, we took advantage of two synthetic procedures developed in our laboratories to carry out both the selenoglycosidation step by using elementary selenium as a cheap selenenating agent [43], and the regioselective installation of the benzyl group at the O-3 position of the sugar [45]. SeDG-Bn proved capable of binding Gal-3^CRD^ and Gal-9N^CRD^ with low micromolar affinity, demonstrating also a higher affinity for both proteins than the SeDG analogue, which lacks the benzyl groups. Encouraged by its good affinity, we performed a biological screening on a selected panel of human cancer cell lines and healthy cells expressing Gal-3^CRD^ or Gal-9N^CRD^. After we verified the non-toxicity of SeDG-Bn, we demonstrated its selective antiproliferative action towards melanoma cells without showing significant cytotoxicity towards normal cells. 

Then, we selected the WM266 cell line to evaluate the effect of our compounds on galectins-mediated activities that lead to cancer progressions [48]. Through a wound healing assay, we showed that cells treated with the SeDG-Bn migrate more slowly than the control and, remarkably, the cell migration induced by Gal-3^CRD^ is specifically abolished by the presence of the examined compound. Moreover, SeDG-Bn inhibits both the melanoma cell invasion of the extracellular matrix and the formation of pseudo-capillaries from endothelial cells, which are two processes that are important for metastatic progression.

Our multidisciplinary approach, encompassing molecular modeling and design, synthetic, and biological chemistry as well as biological characterization, emphasizes the role of synthetic digalactosides as galectin inhibitors in cancer treatment and identified a new molecule with a specific biological activity. 

Moreover, our observations highlight the great potential of galectins as therapeutic targets for the design of selective molecules with enhanced activity.

## Figures and Tables

**Table 1:** Thermodynamic binding parameters of Gal-3^CRD^ and Gal-9N^CRD^ titrated with SeDG and SeDG-Bn at 298 K.

Compound	Kd (μM)	ΔG (kcal/mol)	ΔH (kcal/mol)	−TΔS (kcal/mol)	*n*
**Gal-3^CRD^**					
SeDG	20.2 ± 2.2	−6.31	−4.32 ± 0.27	−1.99	1.09 ± 0.05
SeDG-Bn	2.2 ± 0.4	−7.69	−3.25 ± 0.12	−4.44	1.07 ± 0.03
**Gal-9N^CRD^**					
SeDG	22.4 ± 4.4	−7.10	−5.64 ± 0.36	−1.46	1.01 ± 0.05
SeDG-Bn	2.9 ± 0.3	−7.53	−2.91 ± 0.12	−4.62	0.99 ± 0.03

## Data Availability

The docking data presented in this study will be openly available in our public repository upon acceptance.

## Supplementary Materials

The following supporting information can be downloaded at: www.mdpi.com/article/10.3390/ijms23052581/s1.

## Abbreviations

SeDG	Digalactosyl selenide
SeDG-Bn	3-3′-O-dibenzyl Digalactosyl selenide with
IPTG	Isopropyl β-D-1-thiogalactopyranoside
