# Supplementary material for: Design, Synthesis, and Anticancer Activity of a Selenium-Containing Galectin-3 and Galectin-9N Inhibitor
Source: Int J Mol Sci. 2022 Feb 25;23(5):2581. doi: 10.3390/ijms23052581 (PMC8910629; doi:10.3390/ijms23052581)
Supplement: Supplementary file 1 [file ijms-23-02581-s001.zip › ijms-1581655-supplementary.pdf]

Supporting information to:

Design, Synthesis, and Anticancer Activity of a Selenium-containing Galectin-3 and Galectin-9N Inhibitor

Sonia Di Gaetano, Luciano Pirone, Ioannis Galdadas, Serena Traboni, Alfonso Iadonisi, Emilia Pedone, Michele Saviano, Francesco Luigi Gervasio* and Domenica Capasso *

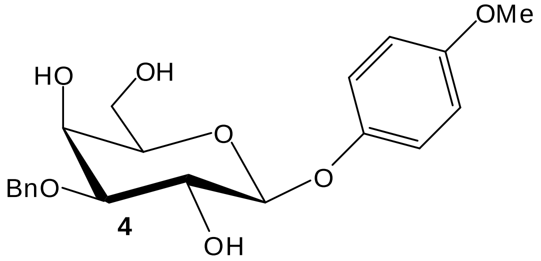

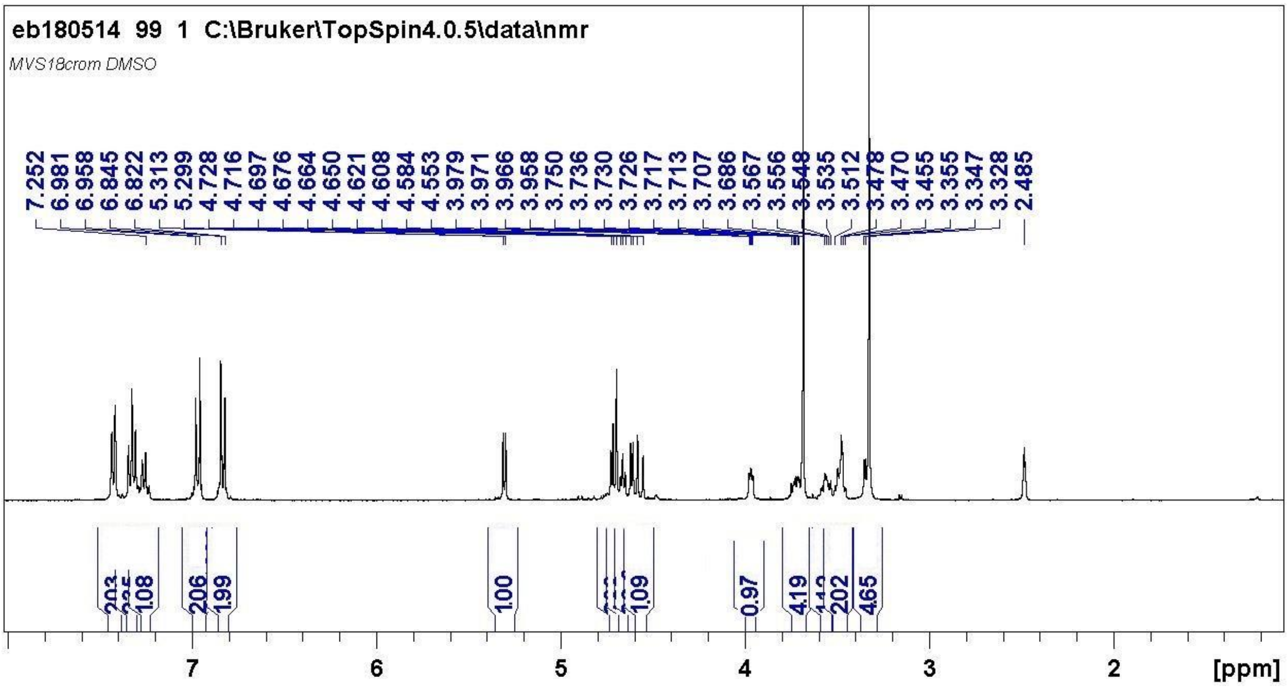

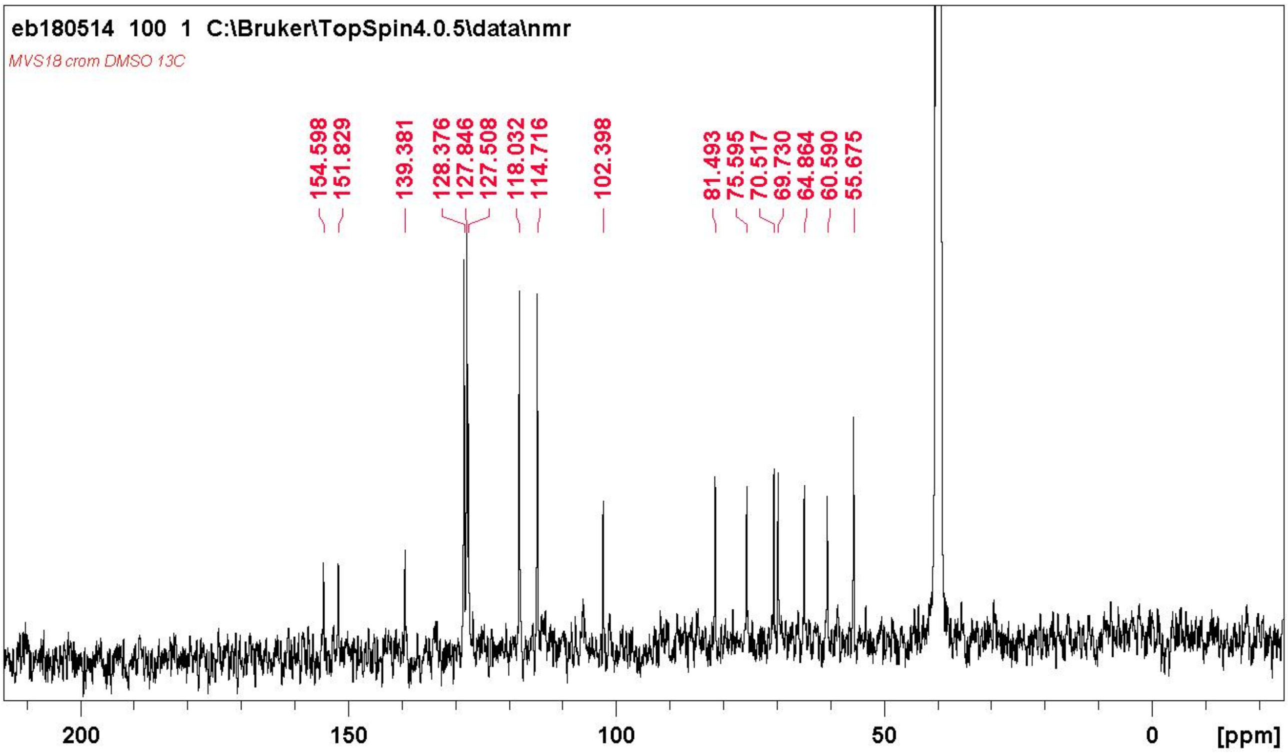
eb180514 100 1 C:\Bruker\TopSpin4.0.5\data\nmr
MVS18 crom DMSO 13C
154.598
151.829
139.381
128.376
127.846
127.508
118.032
114.716
102.398
81.493
75.595
70.517
69.730
64.864
60.590
55.675
200
150
100
50
0
[ppm]

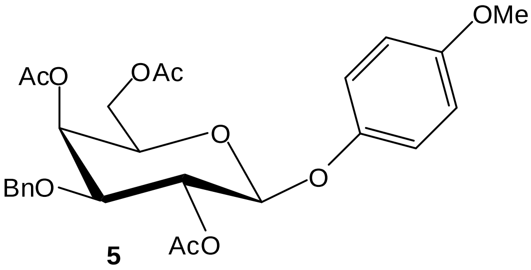
OMe
AcO
OAc
O
BnO
O
AcO

5

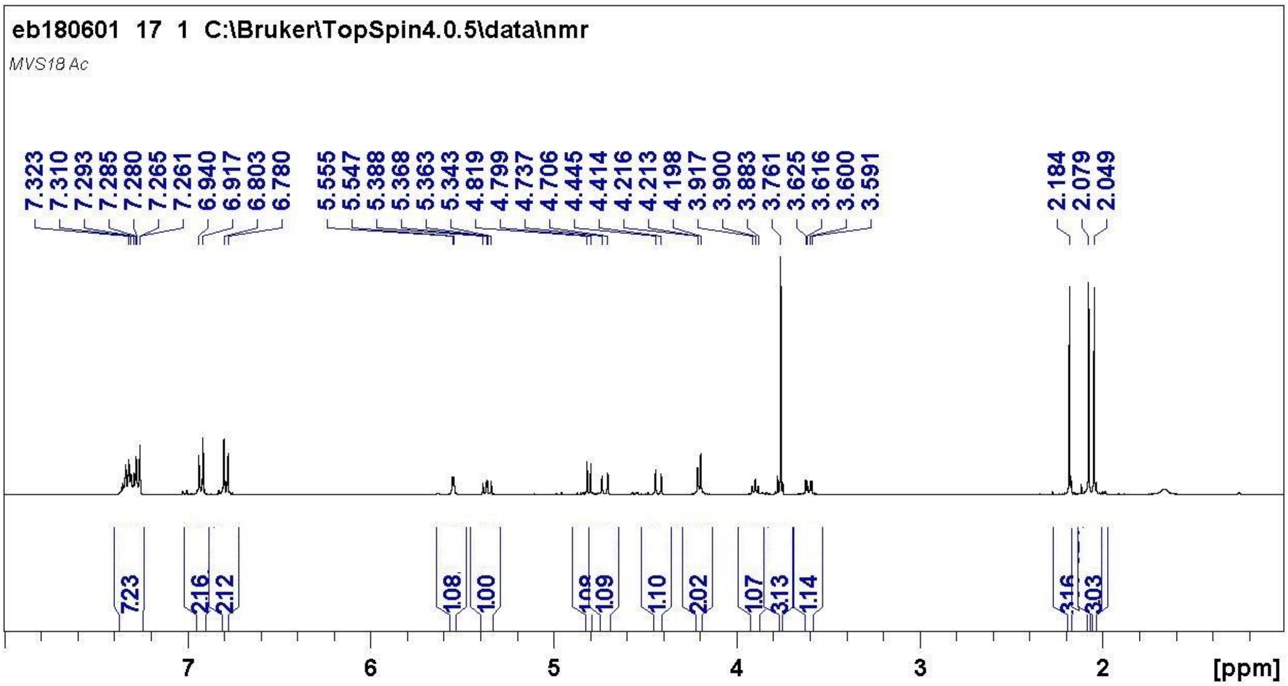
eb180601 17 1 C:\Bruker\TopSpin4.0.5\data\nmr
MVS18 Ac
7.323
7.310
7.293
7.285
7.280
7.265
7.261
6.940
6.917
6.803
6.780
5.555
5.547
5.388
5.368
5.363
5.343
4.819
4.799
4.737
4.706
4.445
4.414
4.216
4.213
4.198
3.917
3.900
3.883
3.761
3.625
3.616
3.600
3.591
2.184
2.079
2.049
7.23
2.16
2.12
1.08
1.00
1.09
1.10
2.02
1.07
3.13
1.14
3.03
7
6
5
4
3
2
[ppm]

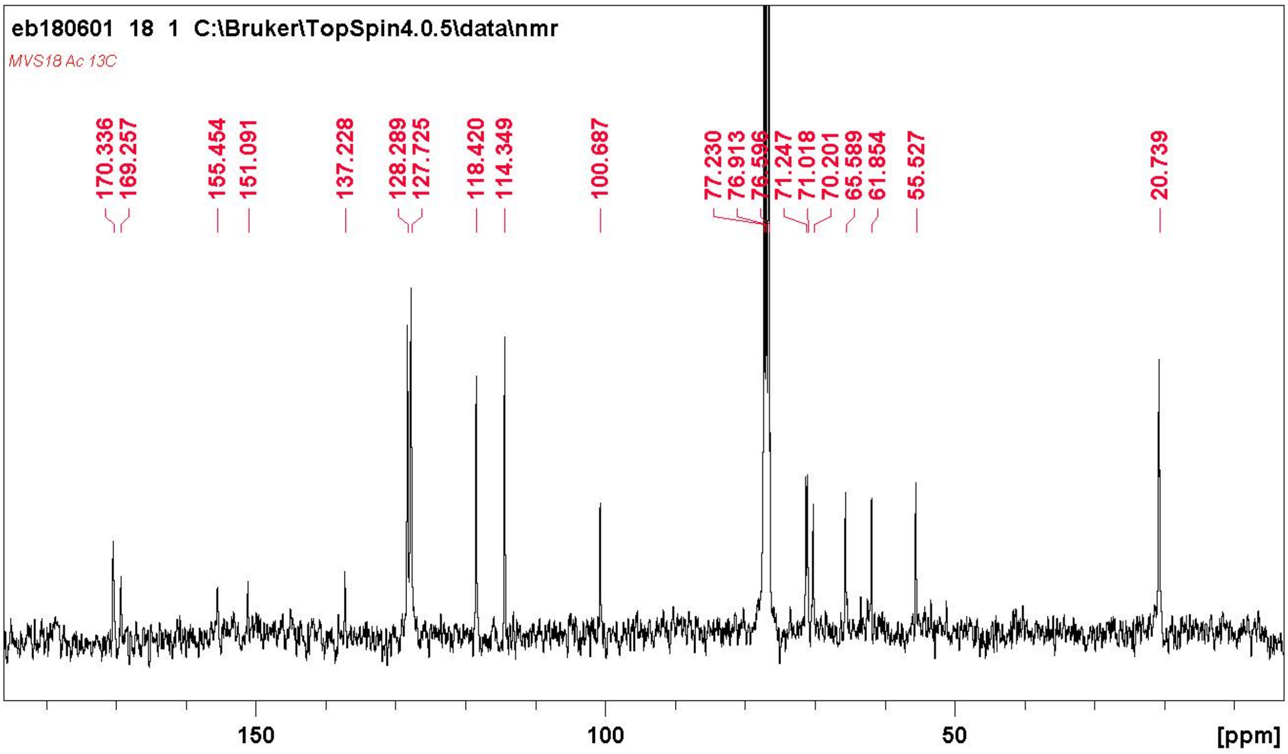

eb180601 18 1 C:\Bruker\TopSpin4.0.5\data\nmr
MVS18 Ac 13C
170.336
169.257
155.454
151.091
137.228
128.289
127.725
118.420
114.349
100.687
77.230
76.913
76.596
71.247
71.018
70.201
65.589
61.854
55.527
20.739
150
100
50
[ppm]

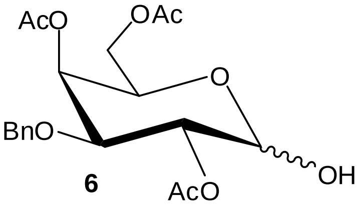

AcO
OAc
O
BnO
AcO
OH

6

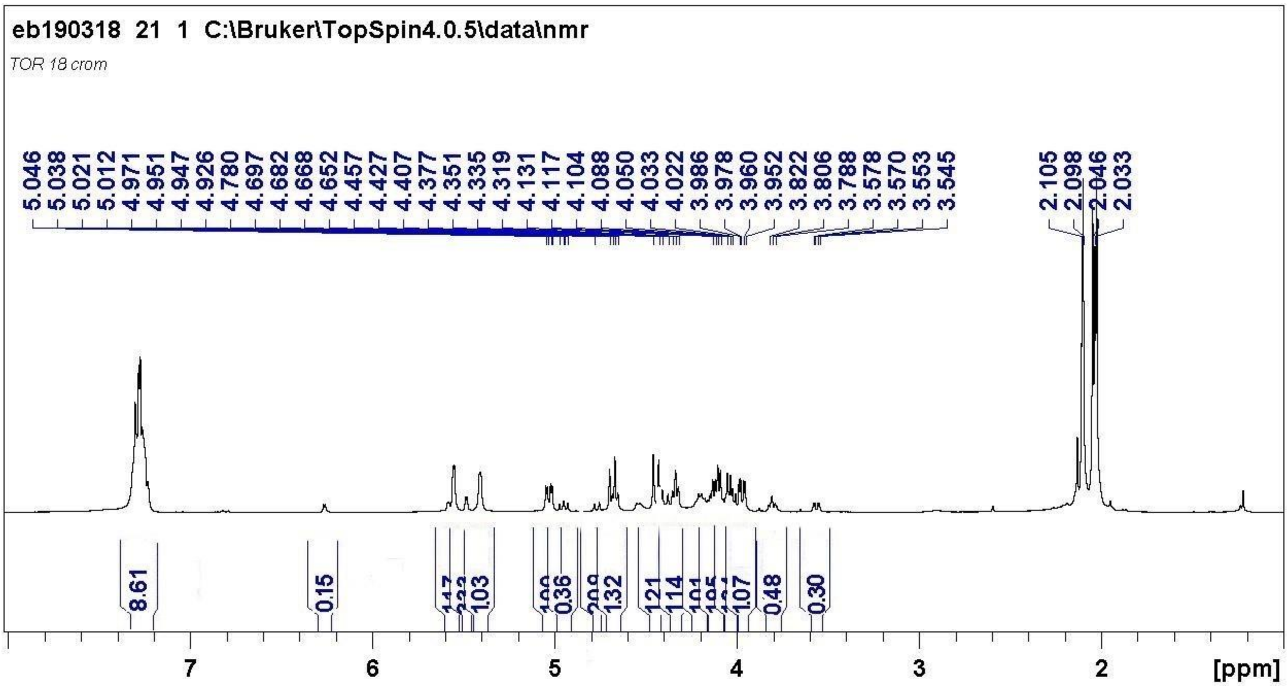

eb190318 21 1 C:\Bruker\TopSpin4.0.5\data\nmr
TOR 18 crom
5.046
5.038
5.021
5.012
4.971
4.951
4.947
4.926
4.780
4.697
4.682
4.668
4.652
4.457
4.427
4.407
4.377
4.351
4.335
4.319
4.131
4.117
4.104
4.088
4.050
4.033
4.022
3.986
3.978
3.960
3.952
3.822
3.806
3.788
3.578
3.570
3.553
3.545
2.105
2.098
2.046
2.033
8.61
0.15
1.03
0.36
1.32
1.21
1.14
1.07
0.48
0.30
7
6
5
4
3
2
[ppm]

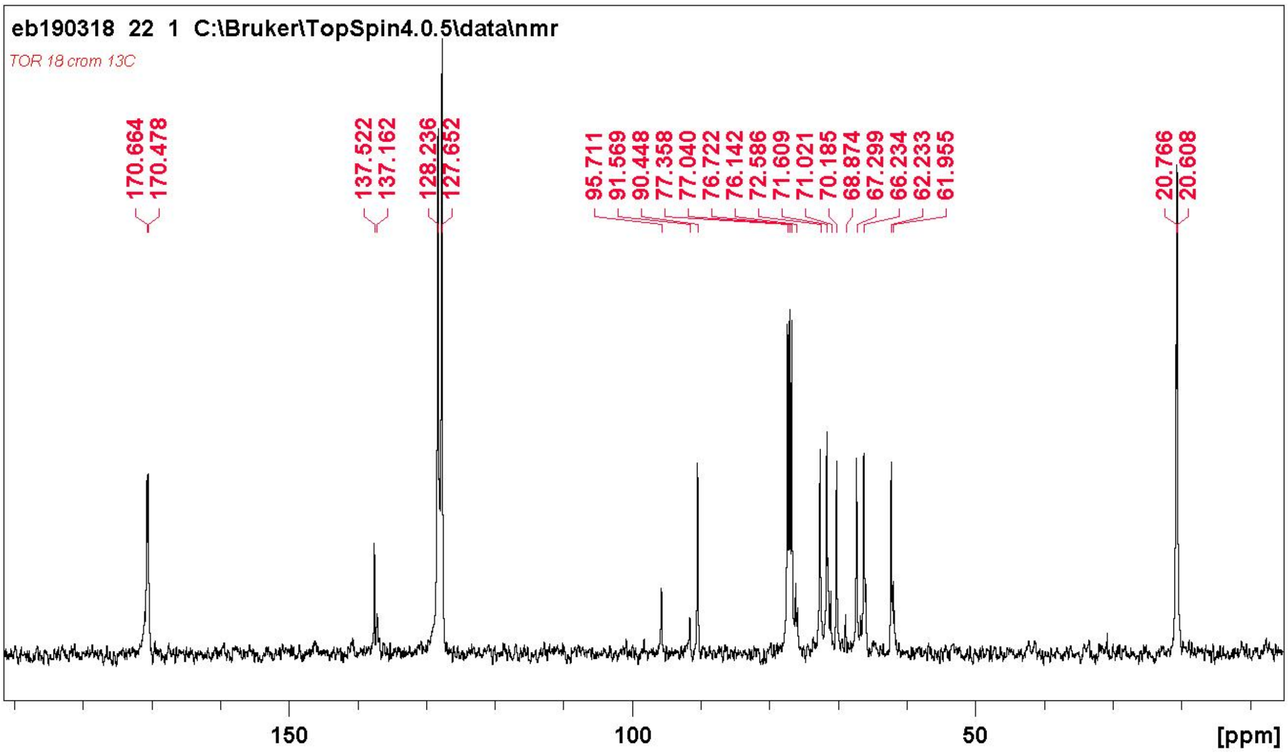
eb190318 22 1 C:\Bruker\TopSpin4.0.5\data\nmr
TOR 18 crom 13C
170.664
170.478
137.522
137.162
128.236
127.652
95.711
91.569
90.448
77.358
77.040
76.722
76.142
72.586
71.609
71.021
70.185
68.874
67.299
66.234
62.233
61.955
20.766
20.608
150
100
50
[ppm]

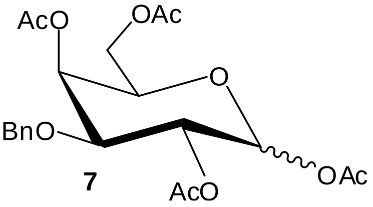
AcO
OAc
O
BnO
AcO
OAc

7

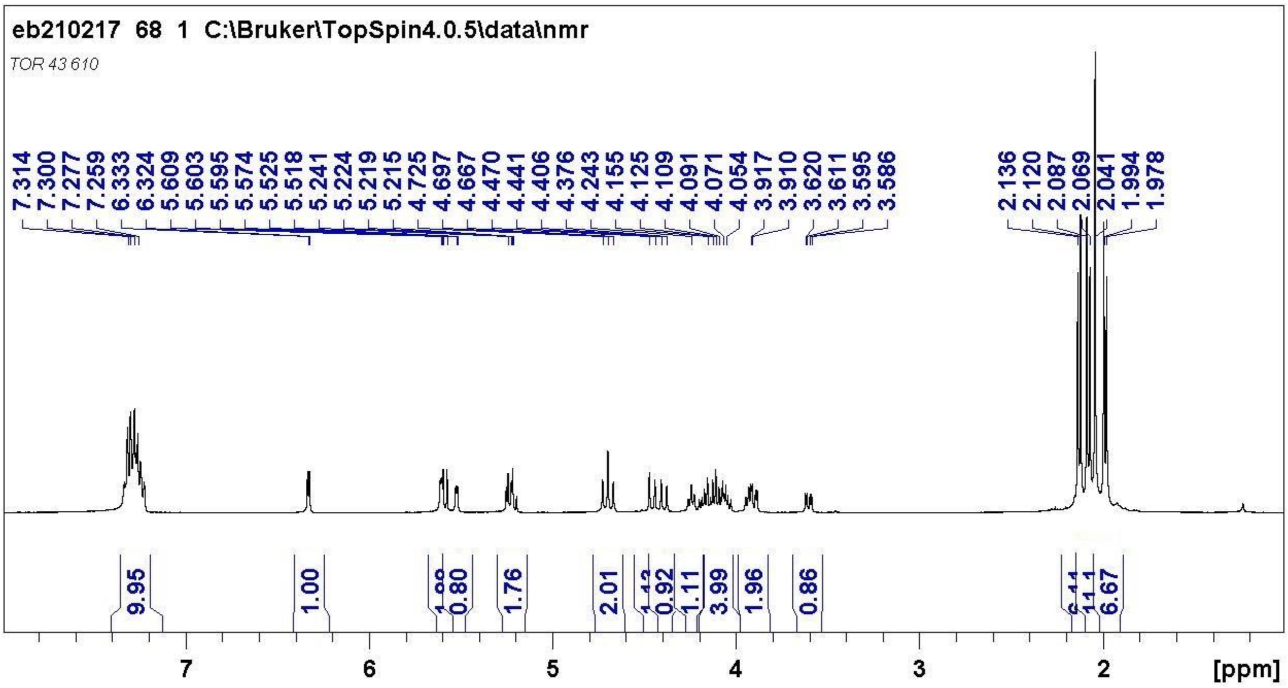
eb210217 68 1 C:\Bruker\TopSpin4.0.5\data\nmr
TOR 43 610
7.314
7.300
7.277
7.259
6.333
6.324
5.609
5.603
5.595
5.574
5.525
5.518
5.241
5.224
5.219
5.215
4.725
4.697
4.667
4.470
4.441
4.406
4.376
4.243
4.155
4.125
4.109
4.091
4.071
4.054
3.917
3.910
3.620
3.611
3.595
3.586
2.136
2.120
2.087
2.069
2.041
1.994
1.978
9.95
1.00
0.80
1.76
2.01
0.92
1.11
3.99
1.96
0.86
6.67
7
6
5
4
3
2
[ppm]

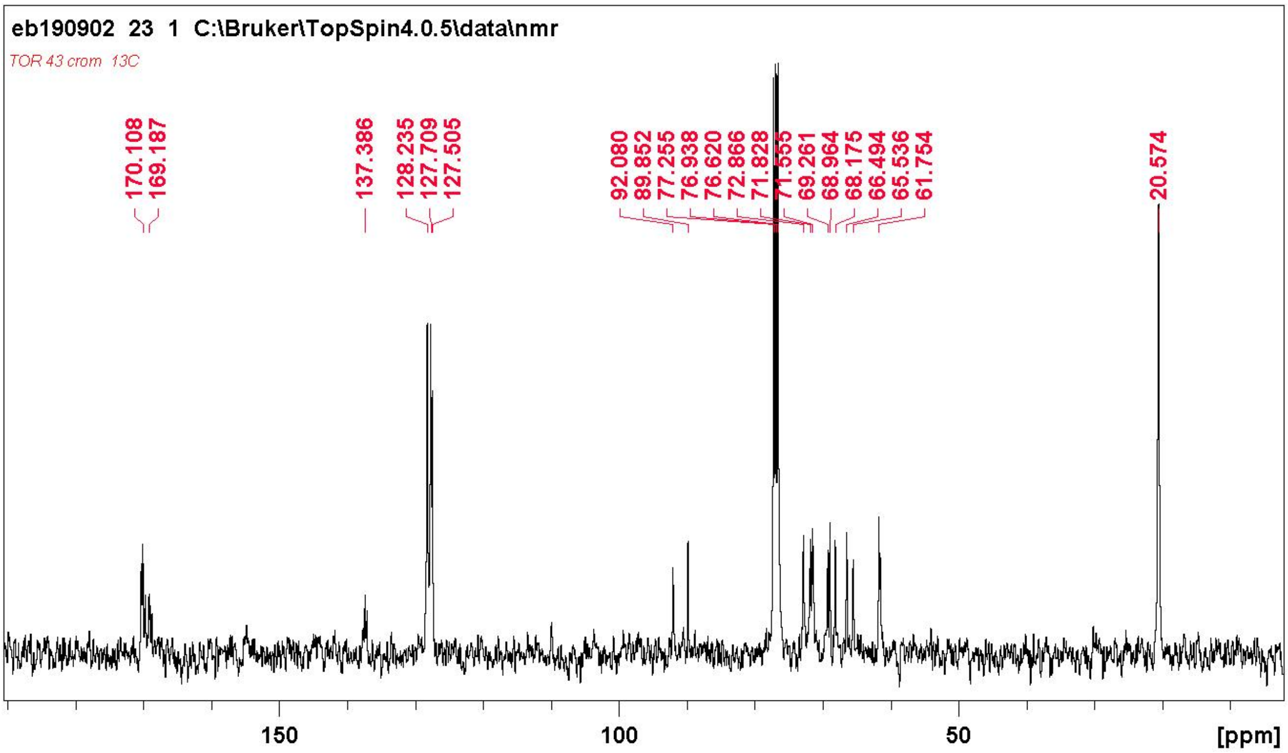
eb190902 23 1 C:\Bruker\TopSpin4.0.5\data\nmr
TOR 43 crom 13C
170.108
169.187
137.386
128.235
127.709
127.505
92.080
89.852
77.255
76.938
76.620
72.866
71.828
71.555
69.261
68.964
68.175
66.494
65.536
61.754
20.574
150
100
50
[ppm]

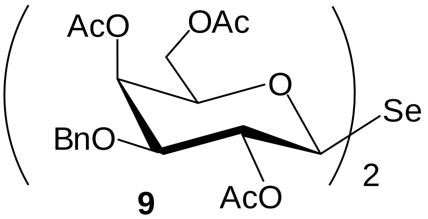
AcO
OAc
O
Se
BnO
AcO
2

9

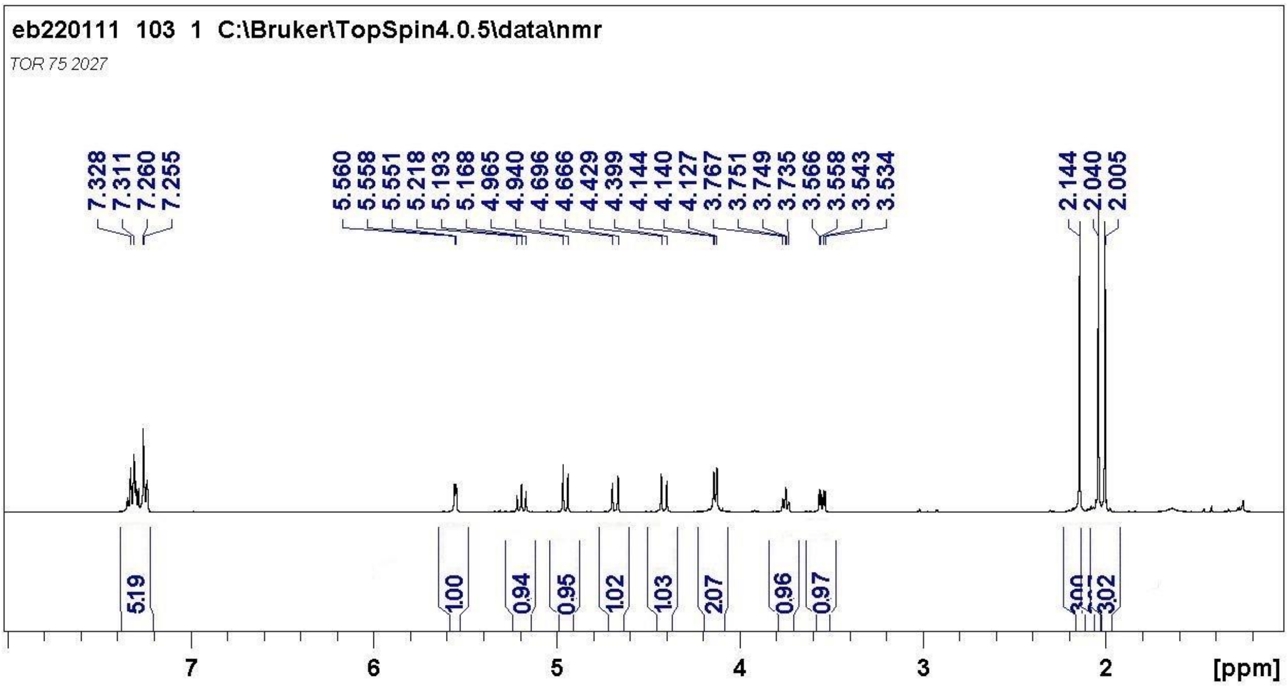
eb220111 103 1 C:\Bruker\TopSpin4.0.5\data\nmr
TOR 75 2027
7.328
7.311
7.260
7.255
5.560
5.558
5.551
5.218
5.193
5.168
4.965
4.940
4.696
4.666
4.429
4.399
4.144
4.140
4.127
3.767
3.751
3.749
3.735
3.566
3.558
3.543
3.534
2.144
2.040
2.005
5.19
1.00
0.94
0.95
1.02
1.03
2.07
0.96
0.97
3.02
7
6
5
4
3
2
[ppm]

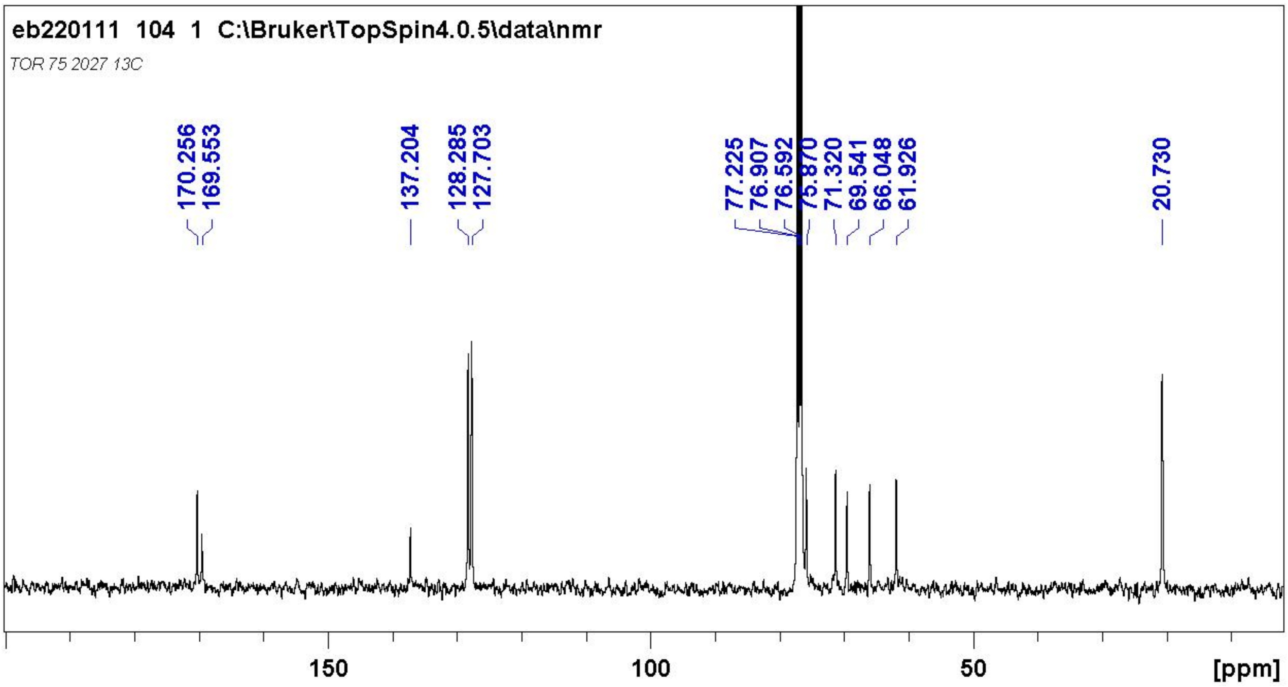
eb220111 104 1 C:\Bruker\TopSpin4.0.5\data\nmr
TOR 75 2027 13C
170.256
169.553
137.204
128.285
127.703
77.225
76.907
76.592
75.870
71.320
69.541
66.048
61.926
20.730
150
100
50
[ppm]

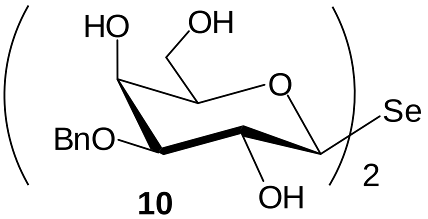
HO
OH
O
Se
BnO
OH
2

10

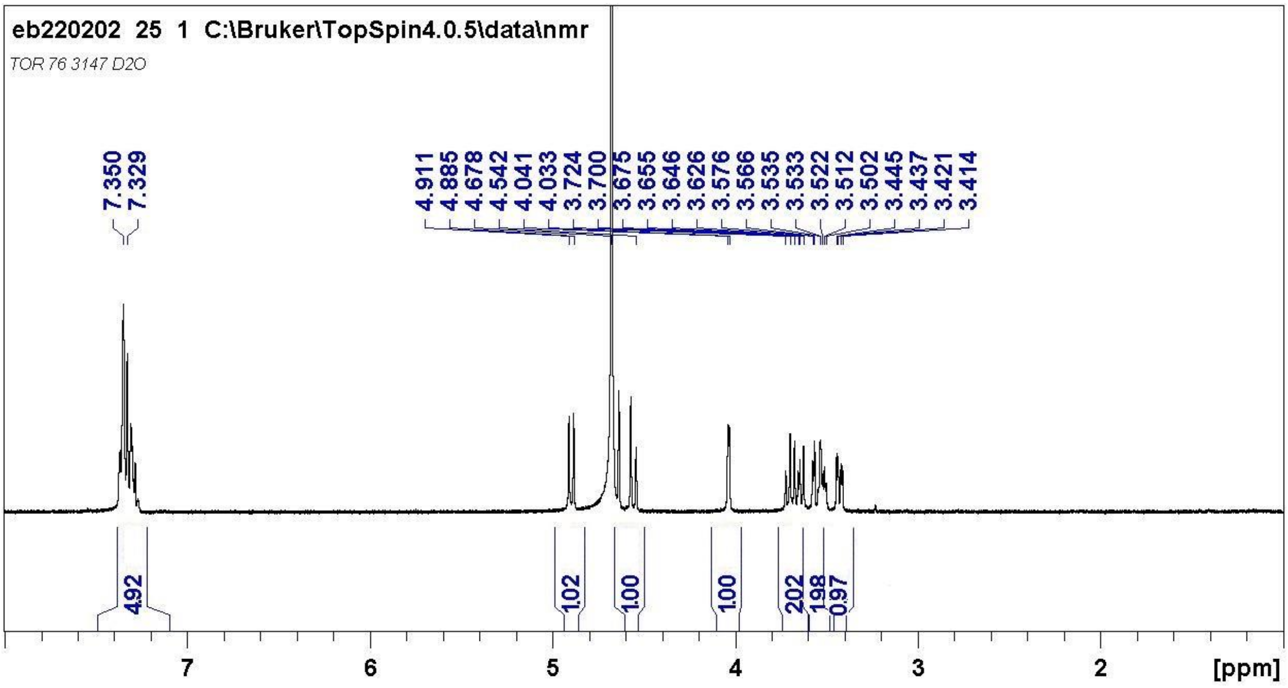
eb220202 25 1 C:\Bruker\TopSpin4.0.5\data\nmr
TOR 76 3147 D2O
7.350
7.329
4.911
4.885
4.678
4.542
4.041
4.033
3.724
3.700
3.675
3.655
3.646
3.626
3.576
3.566
3.535
3.533
3.522
3.512
3.502
3.445
3.437
3.421
3.414
4.92
1.02
1.00
1.00
2.02
1.98
0.97
7
6
5
4
3
2
[ppm]

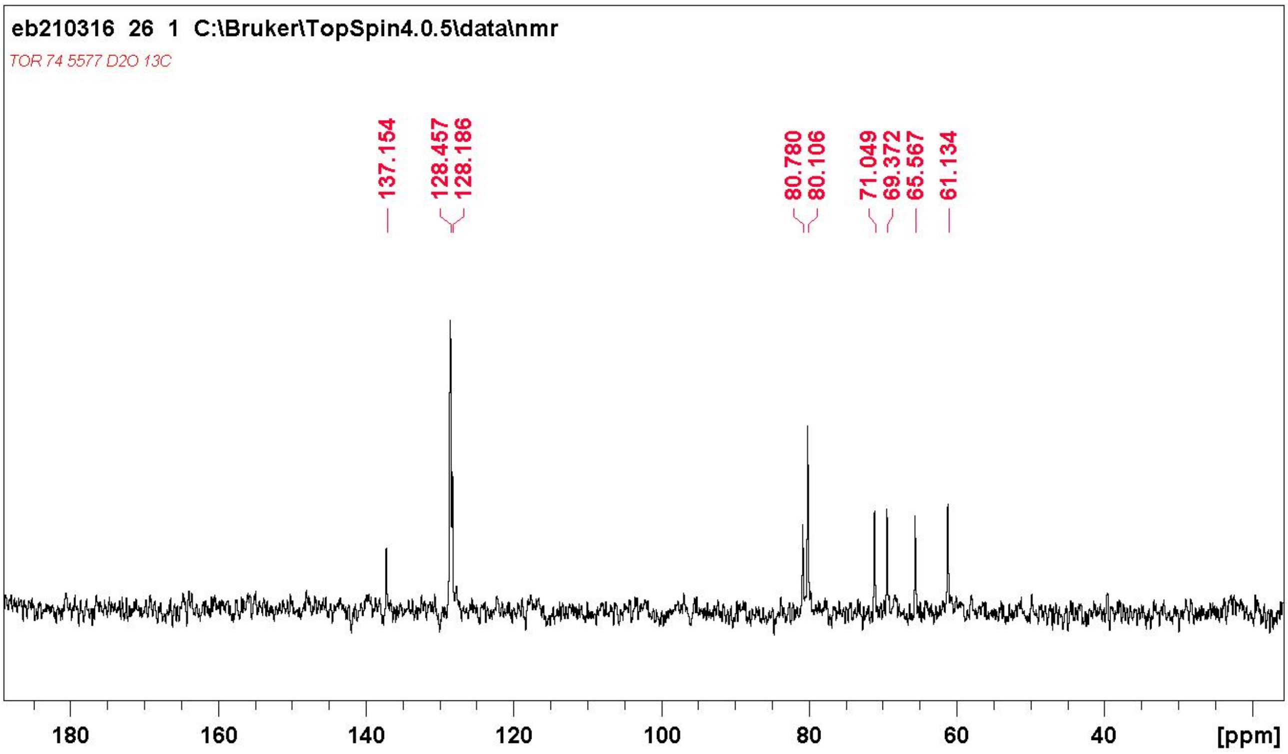

Figure S1. NMR characterization of the synthesized product

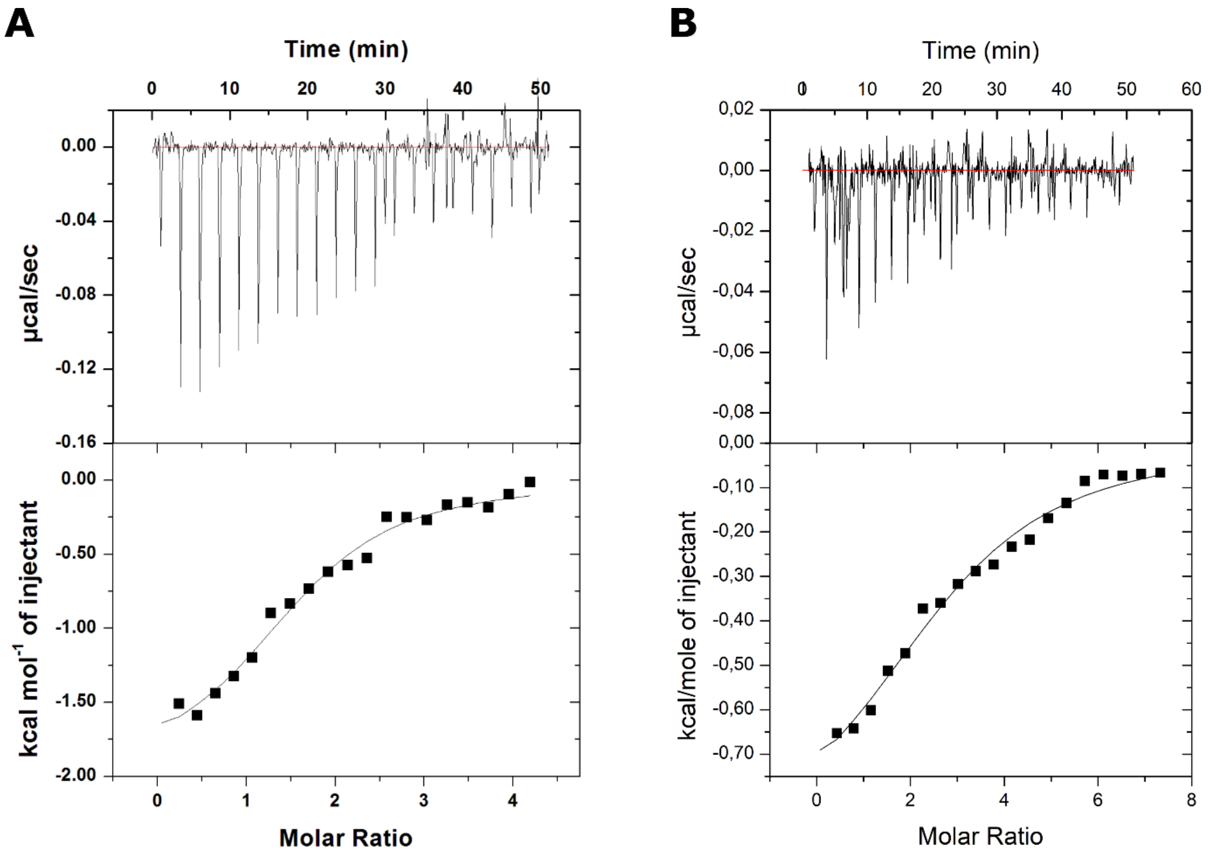

Figure S2. ITC binding studies of Gal3CRD and Gal9-NCRD integrity. Titration of Gal3CRD (A) and of Gal9-NCRD (B) with lactose.

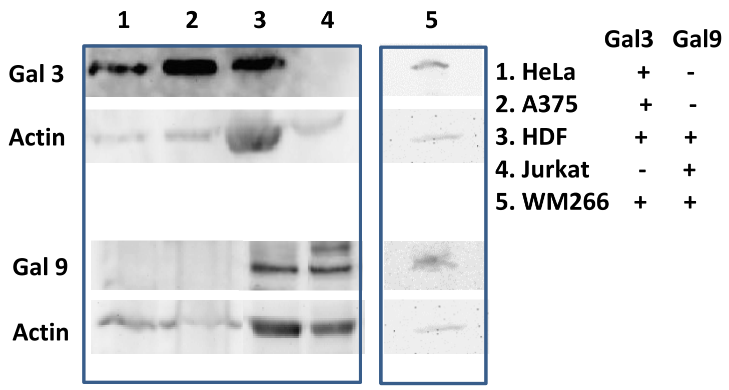

Figure S3. Evaluation of the expression level of Gal-3 and Gal-9 by western blot analyses on different cell lines
